# Burden of disease attributable to PM_2.5_ at low exposure levels: impact of methodological choices

**DOI:** 10.1186/s12940-025-01250-y

**Published:** 2025-12-11

**Authors:** Heli Lehtomäki, Gunn Marit Aasvang, Gerhard Sulo, Bruce R Denby, Otto Olavi Hänninen, Michael Brauer, Gavin Pereira, Omid Dadras, Anette Kocbach Bølling

**Affiliations:** 1 Department of Public Health Solutions, Finnish Institute for Health and Welfare, Helsinki, Finland; 2 Faculty of Health Sciences, University of Eastern Finland, Kuopio, Finland; 3 Department of Air Quality and Noise, Norwegian Institute of Public Health, Lovisenberggata 8, 0456 Oslo, Norway; 4 Department of Global Public Health and Primary Care, University of Bergen, Bergen, Norway; 5 Division for Climate Modelling and Air Pollution, Norwegian Meteorological Institute, Oslo, Norway; 6 Institute for Health Metrics and Evaluation, University of Washington, Seattle, WA United States of America; 7 School of Population and Public Health, University of British Columbia, Vancouver, BC Canada; 8 School of Population Health, Curtin University, Bentley, WA Australia; 9 Centre for Fertility and Health, Norwegian Institute of Public Health, Oslo, Norway; 10 Iranian Research Center for HIV/AIDS (IRCHA), Tehran University of Medical Sciences, Tehran, Iran

**Keywords:** Fine particulate matter, Burden of disease, Uncertainty analysis, Health risk assessment, Cause of death

## Abstract

**Background:**

Exposure to fine particles (PM_2.5_) has been associated with adverse health outcomes, even at low exposure levels (< 10 µg/m^3^). Burden of disease assessments can quantify these associations; however, their sensitivity to methodological choices limits comparability between studies.

**Methods:**

This study aimed to quantify the impact of methodological choices on disease burden attributable to low levels of ambient PM_2.5_, using Norway as a case study. Key methodological choices included (i) population exposure data, (ii) concentration-response curves, and (iii) population health data. Data from national and international sources were applied, including the global burden of disease (GBD) study. Attributable mortality and disability-adjusted life years (DALY) were estimated using burden of disease methodology. Additionally, the impact of choices related to concentration-response curves was assessed for higher exposure levels, using a scenario where exposure distributions were shifted to mean exposures up to 30 µg/m^3^.

**Results:**

Methodological choices related to the concentration-response curves had the largest impacts on the estimated attributable deaths, ranging from − 91% to 104% change relative to the reference estimate (1,448 deaths, 95% CI 502–1497). These choices had a smaller impact on higher exposure levels, varying from − 46% to 53%. The choice of exposure and population health data led to 40% differences in attributable death estimates. DALYs attributable to PM_2.5_ were predominantly driven by years of life lost (YLL: 74%). The choice of relative risk (RR) for the concentration response curve caused around 30% variation in DALY estimates relative to the reference (11,730 DALYs; 5,980 − 16,790), with larger differences for ischemic heart disease (-44 to 79%).

**Conclusion:**

Attributable burden estimates for PM_2.5_ are highly sensitive to key methodological choices, particularly at low exposure levels. Consequently, transparent reporting of the methodological choices and data sources in PM_2.5_ health risk assessments are required to improve comparability and facilitate interpretations of the burden estimates.

**Supplementary Information:**

The online version contains supplementary material available at 10.1186/s12940-025-01250-y.

## Background

 Fine particulate matter (PM_2.5_) has been recognized as the most important environmental risk factor worldwide, contributing to an estimated 4.7 million deaths in 2021 [1]. The strongest associations have been reported for mortality and morbidity due to ischemic heart disease (IHD), stroke, lung cancer, chronic obstructive pulmonary disease (COPD), respiratory infections, and all-cause mortality [2].

Recent studies have increasingly shown that even low levels of PM_2.5_ exposure are associated with an increased risk of adverse health outcomes, with some suggesting higher risk estimates at low levels [3–9]. For example, Brauer et al. [4] reported that exposures as low as 2.5 µg/m^3^ of PM_2.5_ increased the mortality risk, while Chen & Hoek [2] reported higher relative risk estimates when analyses were restricted to low exposure levels. Similarly, the European ELAPSE study found that the hazard ratio for the association between PM_2.5_ exposure and all-cause mortality was more than double for exposures below 12 µg/m^3^, compared to the entire exposure range [6]. In response to the growing evidence of health risks at low PM_2.5_ exposures, the World Health Organization (WHO) recently updated its Air Quality Guidelines (AQG), lowering the recommended PM_2.5_ limit from 10 µg/m^3^ to 5 µg/m^3^ [10]. The U.S. Environmental Protection Agency (US-EPA) also revised the National Ambient Air Quality Standards (NAAQS), reducing the limit from 12 µg/m^3^ to 9 µg/m^3^ [11]. In this paper, low levels refer to concentrations below the WHO and NAAQS thresholds. The European Commission has revised its Ambient Air Quality Directive to align more closely with AQGs, including lowering of PM_2.5_ limit value from 25 µg/m^3^ to 10 µg/m^3^ [12].

Quantifying the health risks associated with air pollution often involves using environmental burden of disease methods to estimate the proportion of disease burden attributable to the exposure [13]. Commonly applied metrics include the number of attributable deaths, diseases, or disability-adjusted life years (DALY) [14]. DALYs quantify the gap between current health status and an ideal health state, combining the years lived with a disease (YLD) and the years of life lost (YLL) due to premature mortality. This method is recommended by WHO and commonly applied by international organizations [15, 16].

Assessing the attributable burden requires data on the population exposure, concentration-response curves, and population health data for the health outcome of interest [13]. Ambient PM_2.5_ concentrations, derived from physical or statistical models with varying spatial resolution, are used to generate exposure distributions or population-weighted means [6, 17–20]. Moreover, the currently applied concentration-response curves for PM_2.5_ range from simple linear curves based on a single risk estimate (e.g. [21][15]) to curves derived from fitting flexible non-linear functions in meta-analyses e.g. an Integrated Exposure-Response (IER) [22], Meta-Regression with Bayesian priors, Regularization and Trimming (MR-BRT) [23], and Global Exposure Mortality Model (GEMM) models [24]. Three key aspects of the concentration-response curves are important to consider when assessing attributable burden: (i) the risk estimate, (ii) the shape of the function, and (iii) the use of a cut-off or a counterfactual level. While mortality data is commonly available from national registries, burden of disease estimates are typically obtained from international sources such as GBD and WHO.

Methodological choices in health risk assessments can have a major impact on the attributable burden of disease. For instance, the number of deaths attributable to PM_2.5_ exposure in total for all Nordic countries varied from 8,500 to 11,400 deaths depending on the health risk assessment tool used [25]. For Switzerland, health risk assessments for ambient air pollution varied widely with a ratio from 0.40 to 2.09 compared to the reference estimate, mainly due to differences in counterfactual scenarios and exposure estimates [26]. Similarly, estimates presented with regular intervals, like the GBD and European Environmental Agency (EEA) estimates, vary due to updates in methods and input data [27, 28]. For instance, the air pollution burden increased by 23% from GBD 2013 to 2015 in low- and middle-income countries due to updated concentration-response curves [29].

The main objective of this paper is to quantify and compare the influence of methodological choices in health risk assessment of PM_2.5_ exposure with a focus on low exposure levels, using Norway as a case study. Specifically, we compare (i) two sources of population exposure data, (ii) a range of concentration-response curves, including choice of shape, risk estimate, and cut-off, and (iii) population health data across three international and national sources. Quantitative assessments of methodological choices and data sources are presented relative to a reference estimate, and for higher exposure levels using a scenario with shifted exposure distributions.

This manuscript was produced as part of the GBD Collaborator Network and in accordance with the GBD Protocol.

## Methods

In this work, we estimated the burden of disease attributable to PM_2.5_ in Norway using an exposure-based approach [13]. The burden of disease attributable to PM_2.5_ (AB) was calculated by multiplying population attributable fraction (PAF) with population burden of disease (BoD) (Eq. 1).

1 $$\mathrm{AB}=\mathrm{PAF}\;\times\;\mathrm{BoD}$$

where BoD refers to different burden of disease metrics, including DALY, YLL, YLD, and number of deaths. The PAF was estimated based on the population exposure data divided into 1 µg/m^3^ intervals (i)(Eq. 2):

2 $$\:\mathrm{P}\mathrm{A}\mathrm{F}=\frac{(\sum\:{(\:RR}_{i}\:\times\:{EF}_{i}\:)-1)}{\left(\sum\:{(\:RR}_{i}\:\times\:{EF}_{i}\:)\right)}$$

where RR_i_ is the relative risk at the exposure level in the interval i and EF_i_ is the fraction of the population exposed to the corresponding PM2.5 level.

When annual population-weighted mean concentration (PWC) of PM_2.5_ was used, the corresponding risk estimate RR_PWC_ was used in the equation above, which is then simplified to (Eq. 3):

3 $$\mathrm{PAF}\;=\frac{{\mathrm{RR}}_{\mathrm{PWC}}-1}{\left({\mathrm{RR}}_{\mathrm{PWC}}\right)}$$

### Exposure data

**Fig. 1:**
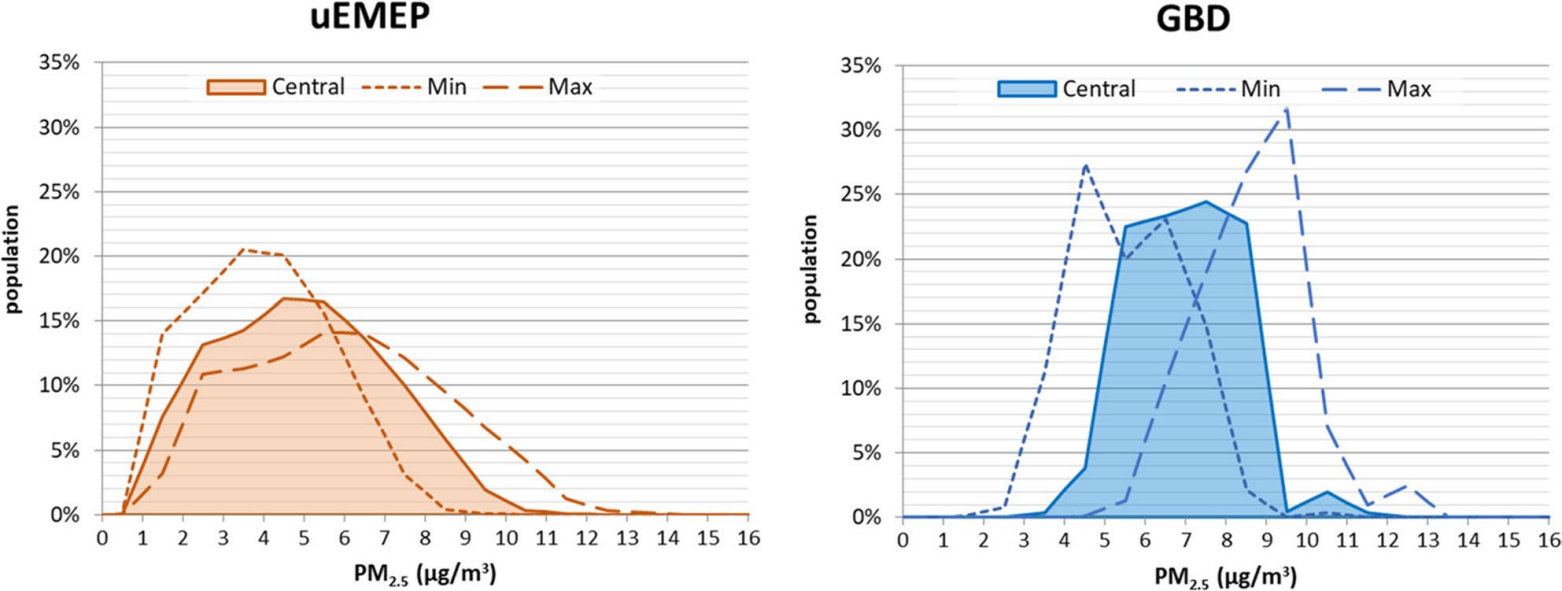
PM_2.5_exposure distributions for Norway 2016 modelled with the uEMEP (population-weighted concentration (PWC) 5.0 µg/m^3^, 95% CI 4.0 to 6.0 µg/m^3^) and GBD (PWC 7.0 µg/m^3^, 95% CI 5.6 to 8.6 µg/m^3^) exposure models. The central exposure distributions for both models are presented with coloured areas, and the uncertainty intervals are presented with dotted and broken lines.

Two exposure models for ambient particulate matter (PM_2.5_) were used, the national Norwegian exposure model uEMEP (urban EMEP), and the global exposure model from the GBD 2017 for the year 2016. The annual population-weighted mean concentrations for PM_2.5_ were estimated at 5.0 µg/m^3^ and 7.0 µg/m^3^ using the uEMEP and GBD models, respectively (Fig. 1). Deaths attributable to PM_2.5_ were estimated for full exposure distributions, with a bin size of 1 µg/m^3^, and using population-weighted mean concentrations (PWC).

The uEMEP is a Gaussian dispersion model used to downscale the chemical transport model EMEP MSC-W (European Monitoring and Evaluation Programme Meteorological Synthesizing Centre West) [30] gridded calculations. The complete model chain for the calculations includes European scale calculations of pollutants at 0.1^o^ x 0.1^o^ resolution, with a nested grid at 2.5 × 2.5 km^2^ covering the Nordic countries from the EMEP MSC-W model. For Norway, uEMEP is applied at 100 × 100 m^2^ resolution. A special methodology ensures no double-counting of the emissions between the EMEP and uEMEP models, and that downscaled emissions are consistent between the two models. Emission sources that are downscaled using uEMEP include road traffic, residential combustion, shipping, and industry. All other sources are modelled using the EMEP MSC-W model. Exposure calculations are made on the 100 × 100 m^2^ uEMEP grid based on aggregated home address data. The model setup and emissions are described in detail in Denby et al. [31]. Calculations using uEMEP are publicly available for all of Norway for the years 2016–2021 [32].

Uncertainty estimates for uEMEP are based on a comparison to measured values in 2016. The uEMEP model shows a −6% bias with a Root mean squared error (RMSE) of 1.2 µg/m^3^ for annual mean PM_2.5_ concentrations. The number of monitoring stations available for this assessment is 27. Annual mean observed and modelled concentrations for all 27 stations are 7.6 and 7.1 µg/m^3^, respectively. The coefficient of determination representing the spatial correlation of the annual mean concentrations is r^2^ = 0.51 [33].

The GBD study uses gridded exposure data for mean annual PM_2.5_ concentration in 0.1˚ x 0.1˚ grids (approximately 11 km x 11 km at the Equator). The exposure estimates are based on satellite aerosol optical depth observations which are related to surface air quality monitor data with global chemical transport model that use data on emissions, chemical reactions, and meteorological conditions to estimate the propagation and concentration of pollutants [34]. For each grid, the model estimates the mean, median and 95% confidence interval for estimated PM_2.5_ concentrations. These gridded exposure data for 2016 for Norway from GBD 2017 were combined with the corresponding gridded population data to generate a population exposure distribution at a resolution of 1 µg/m^3^ with a 95% confidence interval (Fig. 1). The population data was from GBD 2019 for the year 2017.

As the shape and cut-off of the concentration-response curve can influence the BoD estimates depending on the exposure level, we also assessed the impact for exposure distributions with higher PWC. For this purpose, a simplified exposure distribution, with a PWC of 5 µg/m^3^, was shifted to higher concentrations to generate constructed exposure distributions with PWCs of 10, 15 and 30 µg/m^3^ (Supplementary file 1, Figure S1). This shift was achieved by adding 5, 10 or 25 µg/m^3^ to the concentration intervals, while keeping the number of individuals in the interval constant. In this way, the shape of the exposure distribution remained unchanged, while the PWC was shifted to higher concentrations of 10, 15 and 30 µg/m^3^. These shifted exposure distributions reflect a scenario with higher exposure levels.

### Concentration-response curves

The PAF was calculated using a selection of concentration-response curves. These curves included either single relative risk curves, each based on one relative risk estimate from meta-analyses, or multiple relative risk curves derived from fitting non-linear functions for multiple relative risk estimates (Supplementary file 1, Table S1). A literature search was performed to identify large meta-analyses and European multi-center studies reporting relative risks or concentration-response curves for annual PM_2.5_ exposure and mortality from 2010 to July 2021. Only studies presenting data for the whole adult population were included, rather than those reporting only age and sex-specific curves. All studies concerning all-cause mortality, which here refers to mortality due to other causes than injuries or violence, were included. Studies concerning disease-specific mortality were only included if they assessed a set of diseases, to allow for a comprehensive assessment of the BoD attributable to PM_2.5_ exposure. Mortality due to non-communicable diseases and lower respiratory infections (NCD + LRI) is also used as a proxy for all-cause mortality (Supplementary file 1, Table S1). Only European multi-center studies were included as these are considered the most relevant for the Norwegian population. 

**Fig. 2:**
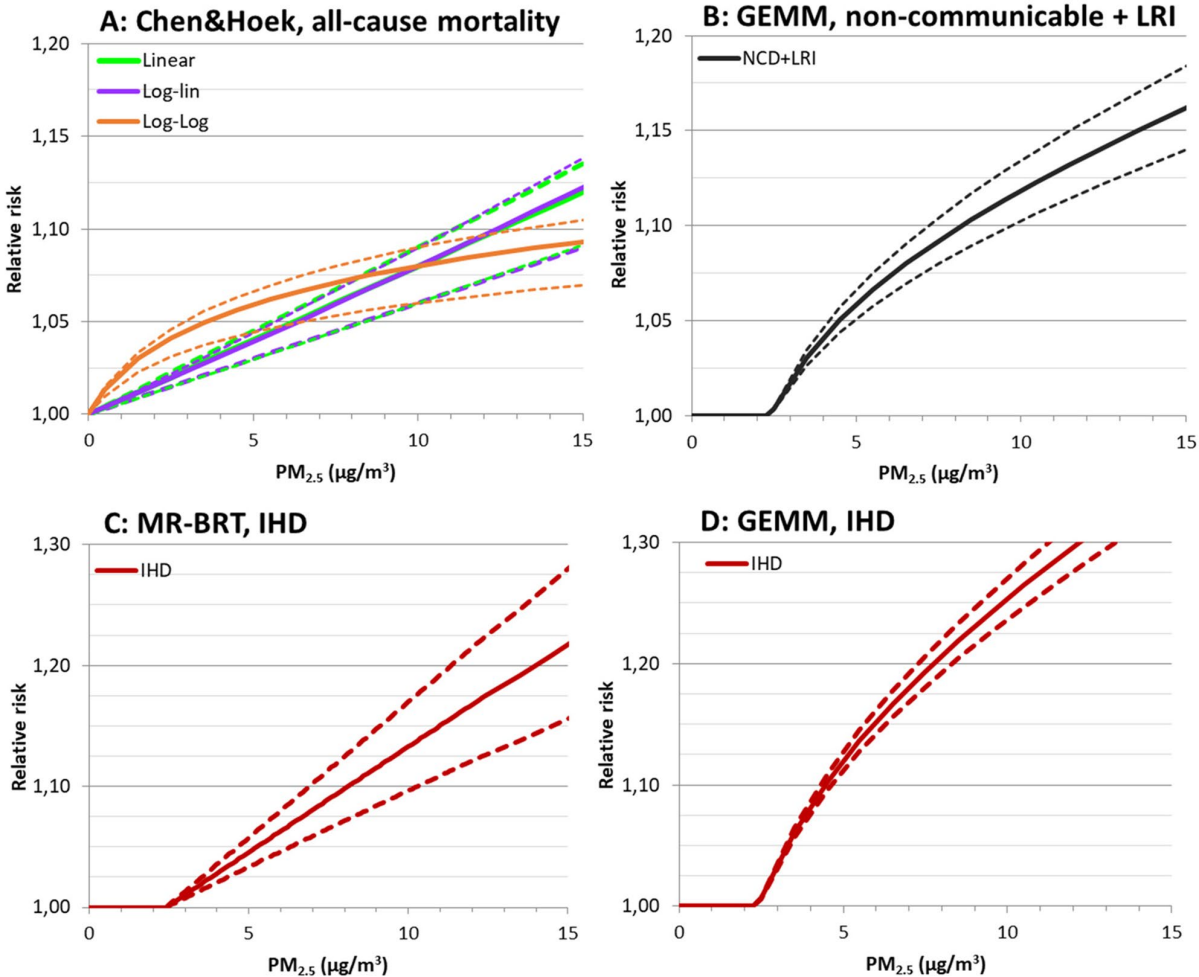
Illustrations of concentration-response curves for PM_2.5_ as relative risk per increment of exposure. Fig. **A**: the three shapes (linear, log-linear, and log-log) of single relative risk curves for all-cause mortality based on relative risk estimates for 10 ug/m^3^ increase in exposure from Chen & Hoek [2]. Fig. **B**: GEMM curve for non-communicable and lower respiratory diseases (NCD + LRI) from Burnett et al. [24]. Fig. **C**: MR-BRT curve for ischemic heart disease (IHD) and Fig **D**: GEMM curve for IHD. Solid lines represent the central estimates and dotted lines 95% confidence intervals of the relative risk.

Curve shapes applied in the literature for single relative risk curves were: linear, log-linear and log-log (Fig. 2 A) [29, 35, 36] (Eq. 4)(Eq. 5)(Eq. 6). These were applied to calculate the relative risk at each exposure level (RR_i_) for the PM_2.5_ concentration in the interval (Ci) based on the RR estimate for a 10 µg/m^3^ increase in PM_2.5_ (RR_10_) and the chosen cut-off (C_0_):

4 $$\mathrm{Linear}: \:{RR}_{i}=\frac{{(RR}_{10}-1)}{10}\left({C}_{i}-{C}_{0}\right)+1$$

5 $$\mathrm{Log-linear}:\:{RR}_i=exp^\beta\:\left(C_i-C_0\right)\;where\;\beta\;=\;\ln\;(RR10)/10$$

6 $$\begin{aligned}&\mathrm{Log-log}:\:{RR}(C_i)=\:\left[\frac{\left(C_i+1\right)}{\left(C_0\:+1\right)}\right]^{\beta\:}where\;\beta\\&=\;\ln(RR_{10})/(\ln(10+C_0+1)-\;\ln(C_0\;+1))\end{aligned}$$

The Global Exposure Mortality Model (GEMM) and the meta-regression—Bayesian, regularized, trimmed tool (MR-BRT) curves are based on non-linear functions that can exhibit a variety of non-linear shapes [23, 24]. Across the entire exposure range, these curves are generally supra-linear, resembling log-log rather than linear or log-linear shapes. Figure 2 C and D show that at low exposures, the MR-BRT curve for IHD is closer to a linear shape, while the GEMM curve exhibits a clear curvature even at very low exposures. The MR-BRT curves used in this study are from the GBD 2021 study.

Cut-off levels of 2, 4, 5 and 6 µg/m^3^ were applied to the concentration-response curves in addition to a curve without any cut-off (the latter is illustrated in Fig. 1a). These cut-offs were chosen based on values commonly used in published burden of disease studies i.e. by IHME GBD studies [16], EEA air quality report [15], and US-EPA [37]. The GEMM functions have a pre-determined cut-off at 2.4 µg/m^3^ [24] (Fig. 2B). For MR-BRT, the cut-off ranges from 2.4 to 5.9 µg/m^3^. In this study, we applied a cut-off of 2.4 µg/m^3^ for MR-BRT curves to facilitate comparison with the GEMM functions.

### Population health data

The population health data used in this study include: (i) all-cause mortality, referring to causes of deaths due to other causes than injuries and violent causes, (ii) mortality due to non-communicable diseases (NCD), and (iii) mortality due to six specific causes: ischemic heart disease (IHD), stroke, chronic obstructive pulmonary disease (COPD), acute lower respiratory infections (ALRI), lung cancer, and type 2 diabetes mellitus (Supplementary file 1, Table S2). The data were focused on adults over 25 years, as the GEMM curves were based on this age group. While some of the relative risk estimates were based on studies of adults over 30 years, the difference in the number of deaths between these two age groups was negligible (< 0.2%). Three sources of population health data were used:

(i) The Global burden of disease (GBD) data by the Institute of Health Metrics (IHME) for Norway for 2016 were downloaded from the GBD results tool GHDx [38] from the GBD 2019 iteration. The GBD study applies a five-step procedure to process input data from the national cause of death registries, including redistribution of garbage-coded deaths [39].
(ii) The WHO global health estimates were downloaded from the website for all-cause and disease-specific mortality [40].
(iii) The Norwegian Cause of Death Registry provided data on disease-specific deaths for Norway in 2016, requested based on the ICD-10 codes definitions used by GBD and WHO (Supplementary file 2, Table S6). In addition, all-cause mortality data were extracted from the Norwegian Cause of Death Registry database [41].

Since disability-adjusted life years (DALYs) capture the total burden of disease better than the number of deaths, DALYs attributable to PM_2.5_ were also estimated in addition to the number of deaths. Estimates were generated for specific diseases to account for the morbidity component (YLD) in addition to mortality (YLL). For the estimation of burden of disease in DALYs, YLLs and YLDs, only data from GBD 2019 were included (Supplementary file 1, Table S3). GBD data were chosen as the Norwegian Cause of Death Registry only provides mortality data, and WHO burden of disease data are updated less frequently than GBD data.

### Reference for comparison across data sources

**Fig. 3:**
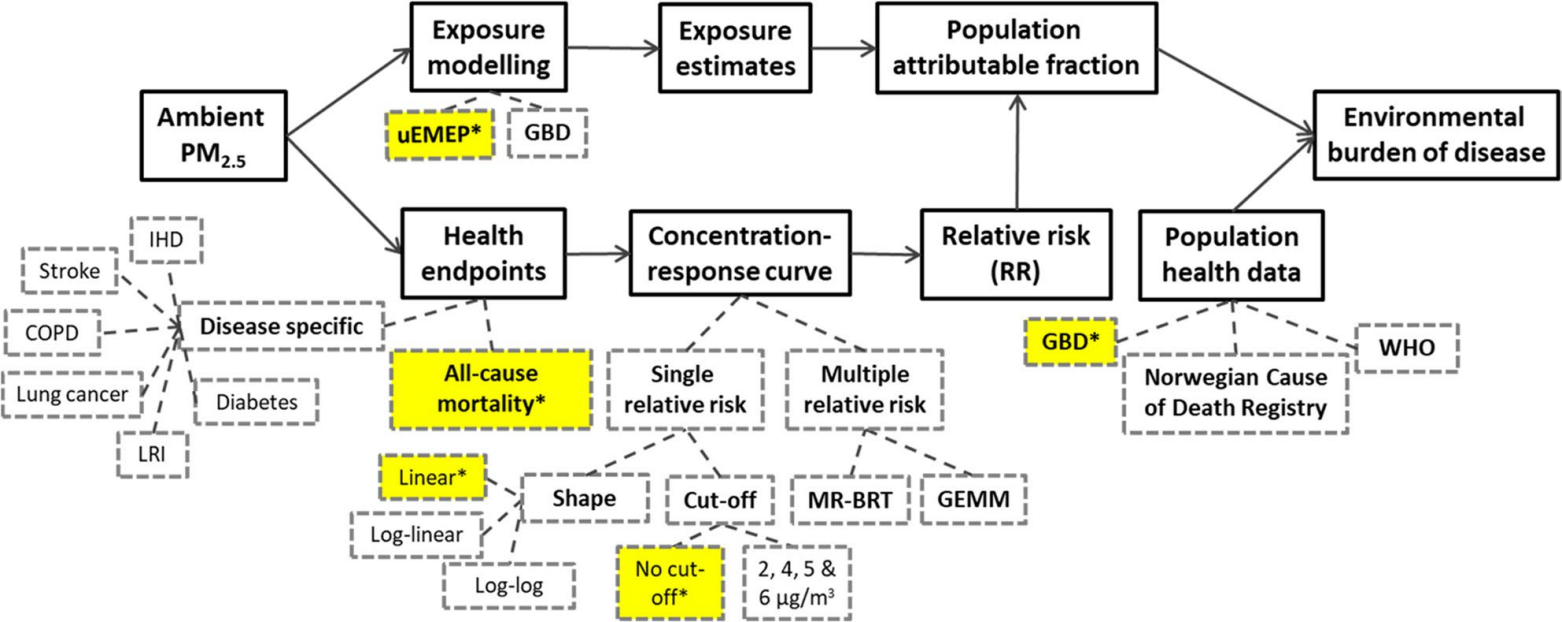
A flow chart of estimating burden of disease attributable to PM_2.5_ exposure. Dashed line boxes represent the methodological choices and data sources included in this study. The yellow boxes with asterix highlight the choices used to obtain the reference estimates

We quantified the impact of methodological choices and data sources by comparing them to a reference estimate, which is based on a specific set of data outlined in Table 1. The methodological choices and data sources included in this study are presented in Fig. 3. Regarding the type of concentration-response curve, the most recent WHO recommendation of using a single relative risk with a linear shape and without a cut-off was chosen as a reference [21]. For the relative risk estimate, we used the updated WHO recommendation, from a recent meta-analysis supporting the 2021 WHO guideline update. This analysis provided a relative risk of 1.08 for all-cause mortality per 10 µg/m^3^ of PM_2.5_ [2]. Accordingly, for disease-specific estimates, concentration-response curves for the reference estimate were also based on relative risks from Chen and Hoek [2] (Supplementary file 1, Table S1).

For population health and exposure data, we selected the best available sources for reference. Since the GBD study provides high-quality national estimates for mortality and other burden of disease metrics, the GBD 2019 data for Norway for 2016 were used as reference population health data. The Norwegian exposure model uEMEP was used as a reference for exposure as it has the highest spatial resolution and uses updated national input data.

When the non-linear GEMM and MR-BRT curves were included in the comparisons, the single relative risk curves were based on a cut-off at 2.4 µg/m^3^ and the log-log shape.

**Table 1:** Methodological choices and data sources for reference estimates for the burden of disease attributable to PM_2.5_ in this study

Methodological choice/data type for reference estimate:	
Relative risk	1.08 per 10 µg/m^3^ (Chen & Hoek [2])^a^
Concentration-response curve	Linear
Cut-off	No cut-off (0 µg/m^3^)
Exposure model	Norwegian model uEMEP, mean 5.0 µg/m^3^
Population health data	GBD 2019 study for 2016

^a^ The disease specific estimates from Chen and Hoek 2020 listed in table 1 were used as reference input data for the disease specific estimates

### Uncertainty estimation

The uncertainties in the BoD estimates were calculated based on the available 95% CI of the input data, i.e. the relative risk estimate, the population health data, and the exposure distribution. In these calculations, the 2.5% and 97.5% values from the 95% CI were used as input in the BoD calculations instead of the central estimate. This approach allowed for a quantitative comparison of the relative importance of the uncertainty in each input data category for the resulting uncertainty of the BoD estimate. In each section of the results, the 95% CI of the attributable BoD estimates reflects the 95% CI of the input parameter being examined, as described in figure captions. It is important to note that no uncertainty intervals were provided by the Norwegian Cause of Death Registry for their mortality data.

## Results

### Influence of exposure data

**Fig. 4:**
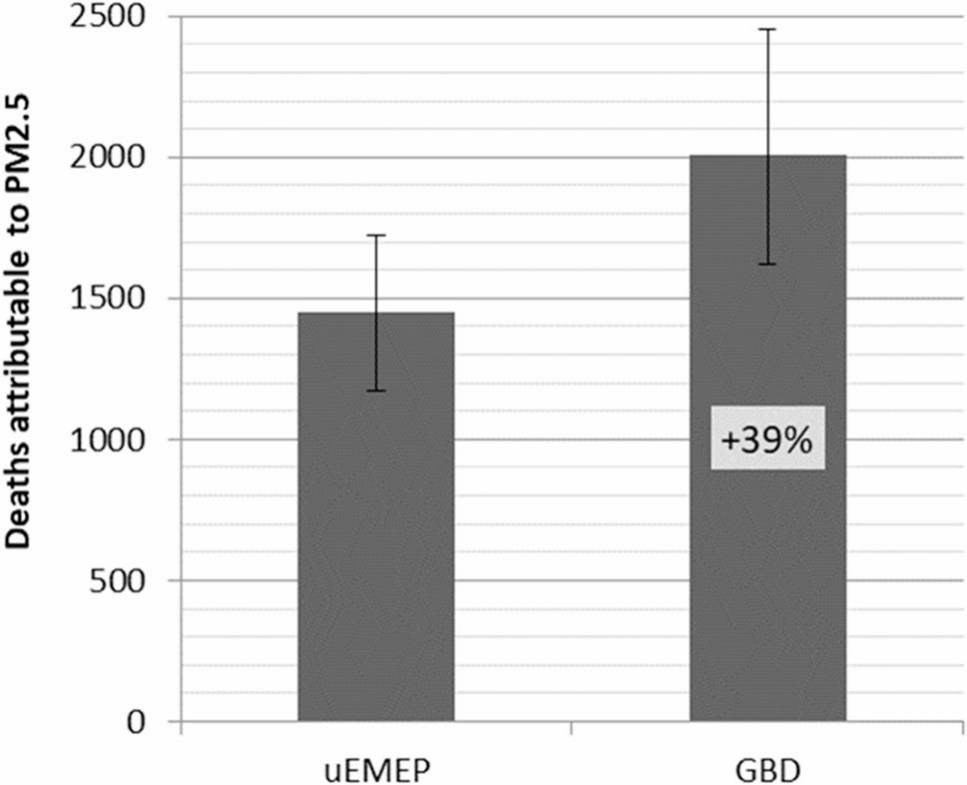
Impact of different sources of exposure data on estimated deaths attributable to PM_2.5_ in Norway 2016. The estimates reflect the national uEMEP model and GBD exposure data with 95% uncertainty intervals.

The reference estimate for the number of deaths attributable to PM_2.5_ was 1,448 (95% CI 1,170 to 1,725) when estimated using the national uEMEP exposure data. The estimate was 39% higher when the exposure distribution from GBD (2,010 deaths, 95% CI 1,620 to 2,454) was used, reflecting the difference between the two exposure distributions (Fig. 1). Note that the uncertainty intervals for the attributable number of deaths, based on the 95% CI of the exposure distributions, were overlapping (Fig. 4).

The application of the mean PWC instead of full exposure distribution resulted in identical estimates of attributable deaths when no cut-offs were applied. However, when a cut-off higher than 2 µg/m^3^ was applied, the use of PWC instead of exposure distribution resulted in considerably lower estimates. Specifically, the estimated number of deaths was 32% and 100% lower than when using the full exposure distribution for a cut-off at 4 and 6 µg/m^3^, respectively. When a log-log concentration-response curve was applied, the difference in attributable deaths compared to full exposure distribution differed with 4%, 8%, −22% and − 100% for cut-offs at 0, 2, 4 and 6 µg/m^3^, respectively.

### Influence of concentration-response curve: relative risk, shape and cut-off

**Fig. 5:**
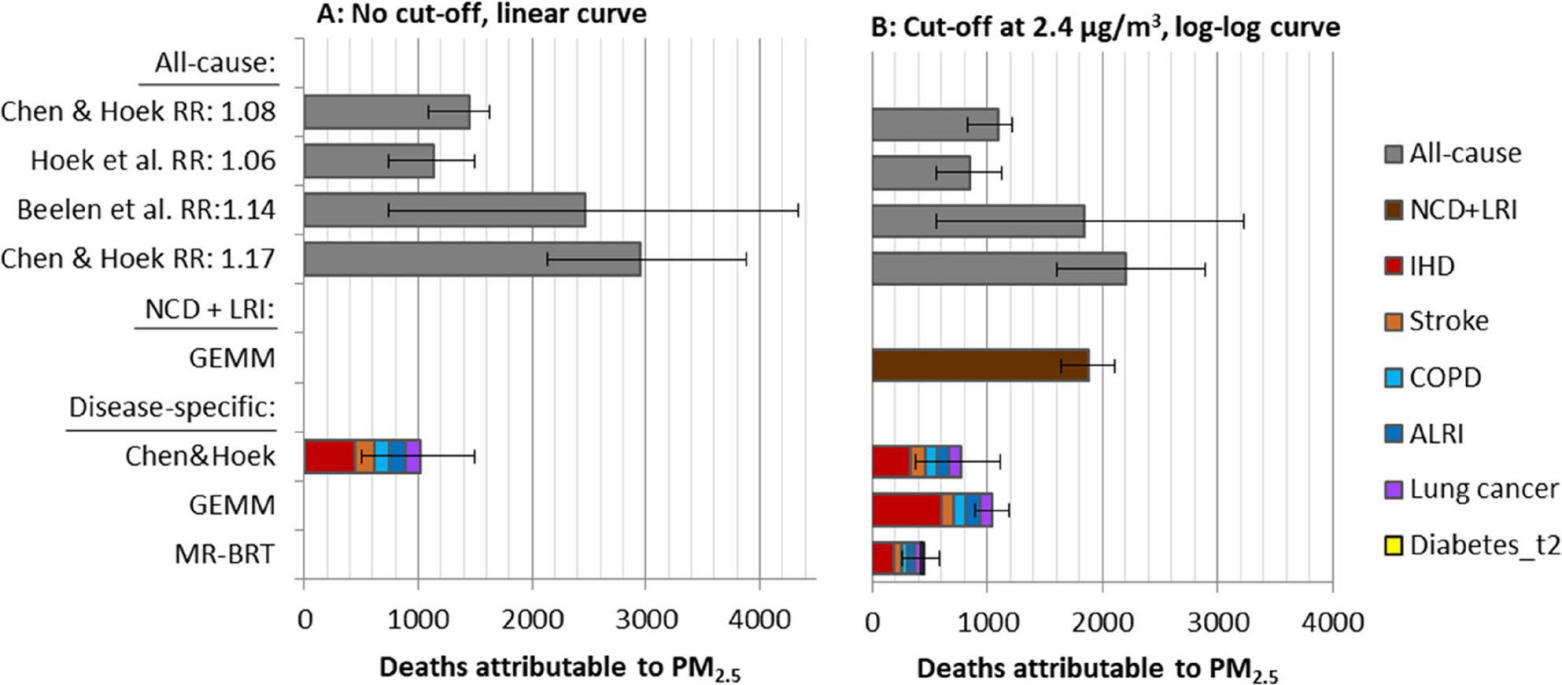
Influence of relative risk (RR) estimates on deaths attributable to PM_2.5_ in Norway in 2016 (PWC 5 µg/m^3^) with 95% CI from relative risks. For the disease specific estimates, uncertainty bars reflect the sum of uncertainties of the individual causes of death. In figure (**A**) linear concentration-response curves with no cut-off is applied. In Figure (**B**), the RRs for all-cause and disease specific mortality were estimated using log-log curve with a cut-off at 2.4 µg/m^3^. NCD+LRI: Non-communicable diseases + lower respiratory.

The relative risk estimate (RR: 1.17) for low exposures (< 10 µg/m^3^) by Chen & Hoek [2] resulted in the highest all-cause mortality estimate (2,950 deaths; 95% CI 2131–3885), which was approximately two times higher than the reference estimate (1,448 deaths; 95% CI 502–1497 from relative risk) (Fig. 5 A). The lowest estimate, based on the previous WHO recommendation (Hoek et al. [42], RR: 1.06), was 22% lower than the reference. There were major differences in the uncertainties based on 95% CI of relative risk estimates (Fig. 5 A). For high exposures (PWC of 30 µg/m^3^) the highest estimate was 53% higher (Beelen et al. [18], RR: 1.14) than the reference estimate of 7342 deaths, and the lowest estimate was 19% lower (Hoek et al. [42], RR: 1.06) than the reference (Supplementary file 1, Figure S2).

The estimated attributable deaths for disease-specific mortality were 29% lower than all-cause mortality when Chen & Hoek [2] disease-specific relative risks were used. Deaths due to ischemic heart disease accounted for the biggest share of disease-specific mortality, with a relative contribution of 44%, while the four other diseases had similar relative contributions. For high exposures (PWC 30 µg/m^3^), disease-specific mortality was 36% lower than the all-cause mortality (Supplementary file 1, Figure S2).

For comparison with the non-linear GEMM and MR-BRT curves with a 2.4 µg/m^3^ cut-off, the relative risks for all-cause and disease-specific mortality were estimated using a log-log curve and a cut-off at 2.4 µg/m^3^. The GEMM NCD + LRI curve resulted in estimates that were approximately 70% higher than the reference all-cause mortality estimate of 1,092 deaths based on Chen & Hoek [2] (Fig. 5B). The sum of attributable deaths from the five disease-specific causes, based on GEMM curves, was about 40% lower than the estimates for the NCD + LRI curve. Finally, the estimates for the five causes of death based on GEMM and MR-BRT functions were 35% higher, and 40% lower, respectively, compared to the reference estimate for cause-specific diseases (769 deaths) (Fig. 5B).

The uncertainties were considerably higher for disease-specific estimates based on Chen & Hoek [2] relative risks compared to GEMM curves, with the highest uncertainty for acute lower respiratory infections (ALRI) and stroke. For high exposures, the reference estimate for disease-specific deaths was 3,004, while GEMM and MR-BRT disease-specific estimates were 80% and 54% higher than the reference (Supplementary file 1, Figure S2). The results from relative risk estimate comparisons for low (PWC 5 µg/m^3^) and high (PWC 30 µg/m^3^) are presented in Supplementary file 1, Table S5.

**Fig. 6:**
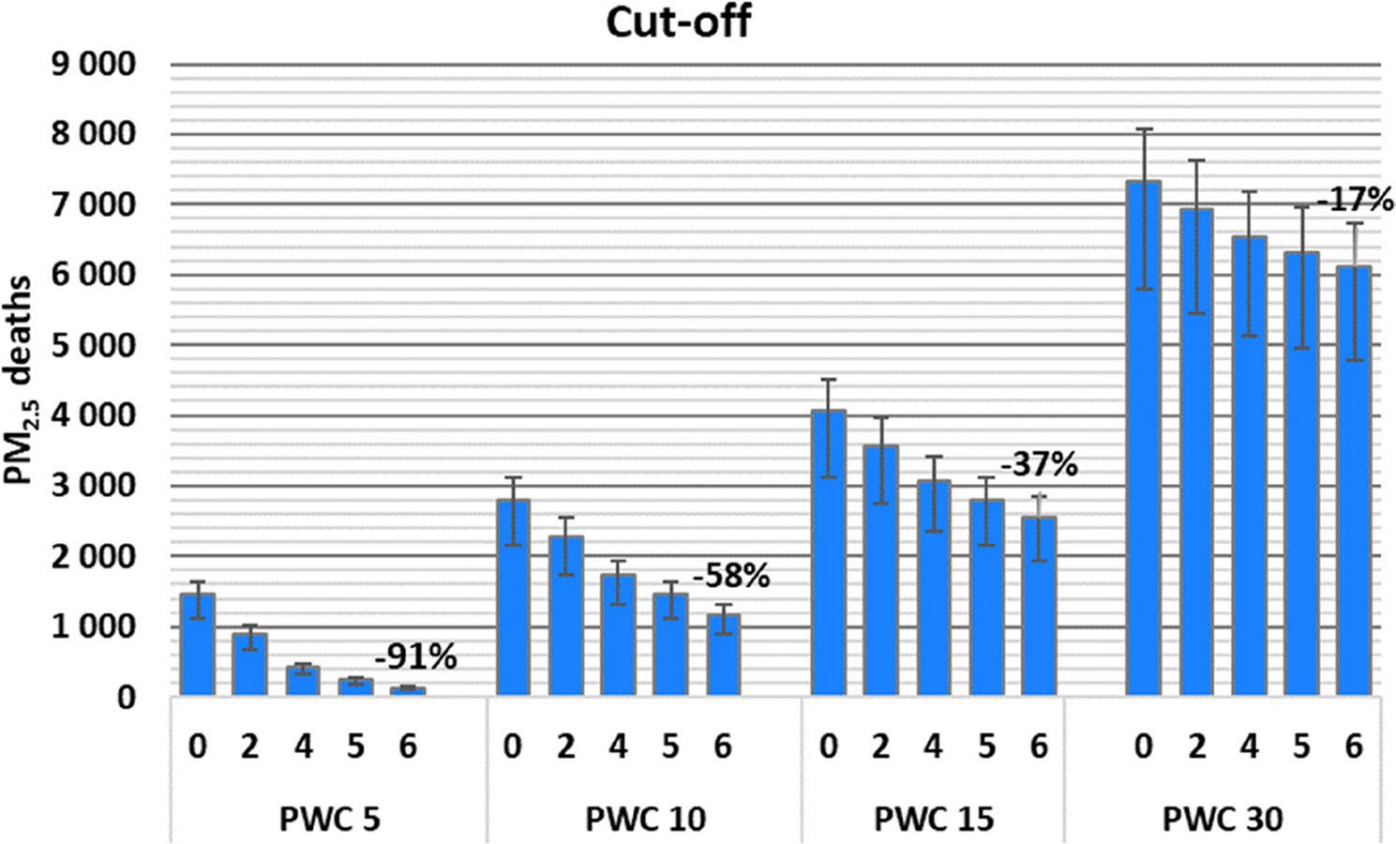
Influence of the five cut-off levels (0, 2, 4, 5 & 6 µg/m^3^) on estimated deaths attributable to PM_2.5_ for exposure levels with population-weighted concentrations (PWC) of 5, 10, 15, and 30 µg/m^3^. Error bars reflect 95% CI from relative risk.

Introducing cut-off levels of 2, 4, 5 and 6 µg/m^3^ in the linear curve reduced the attributable death estimates by about 40%, 70%, 80% and 91%, respectively at low exposures (mean PWC 5 µg/m^3^), in comparison to the curve with no cut-off (Fig. 6). The influence of the cut-off level diminished for exposure distributions with higher mean PWC (Fig. 6). For log-linear and log-log curves, the influence of including cut-offs followed the same pattern as for the linear curve (data not shown).

**Fig. 7:**
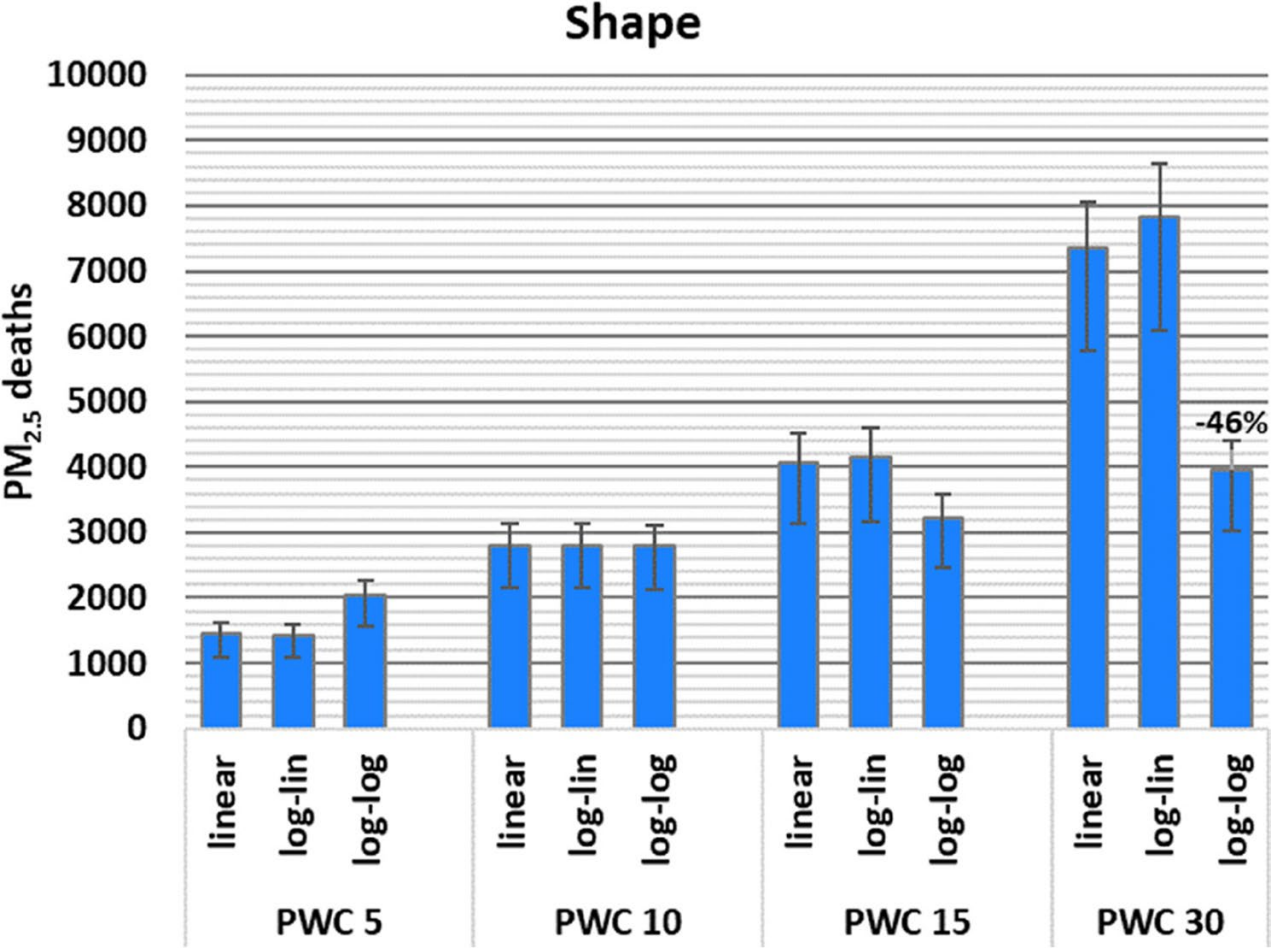
Influence of the three concentration-response curve shapes on estimated deaths attributable to PM_2.5_ for exposure levels with population weighted concentrations (PWC) of 5, 10, 15, and 30 µg/m^3^. Error bars reflect 95% CI of the relative risk.

Regarding the shape of the concentration-response curve, applying a log-linear instead of a linear curve shape had a minor influence on the attributable death estimates, while the log-log shape resulted in larger differences (Fig. 7). For instance, while the log-log shape resulted in a 41% increase in estimated deaths relative to the linear curve at 5 µg/m^3^ mean exposure, the corresponding influence of curve shape was a 21% increase for 15 µg/m^3^, but a 46% decrease at exposure of 30 µg/m^3^. This can be explained by the steeper risk increase of the log-log curve at low exposures, while it levels off at higher exposures. Overall, the influence of introducing a cut-off in the curve is smaller for exposure distributions with higher PWC, while the influence of the curve shape varies depending on the exposure level.

### Influence of population health data

**Fig. 8:**
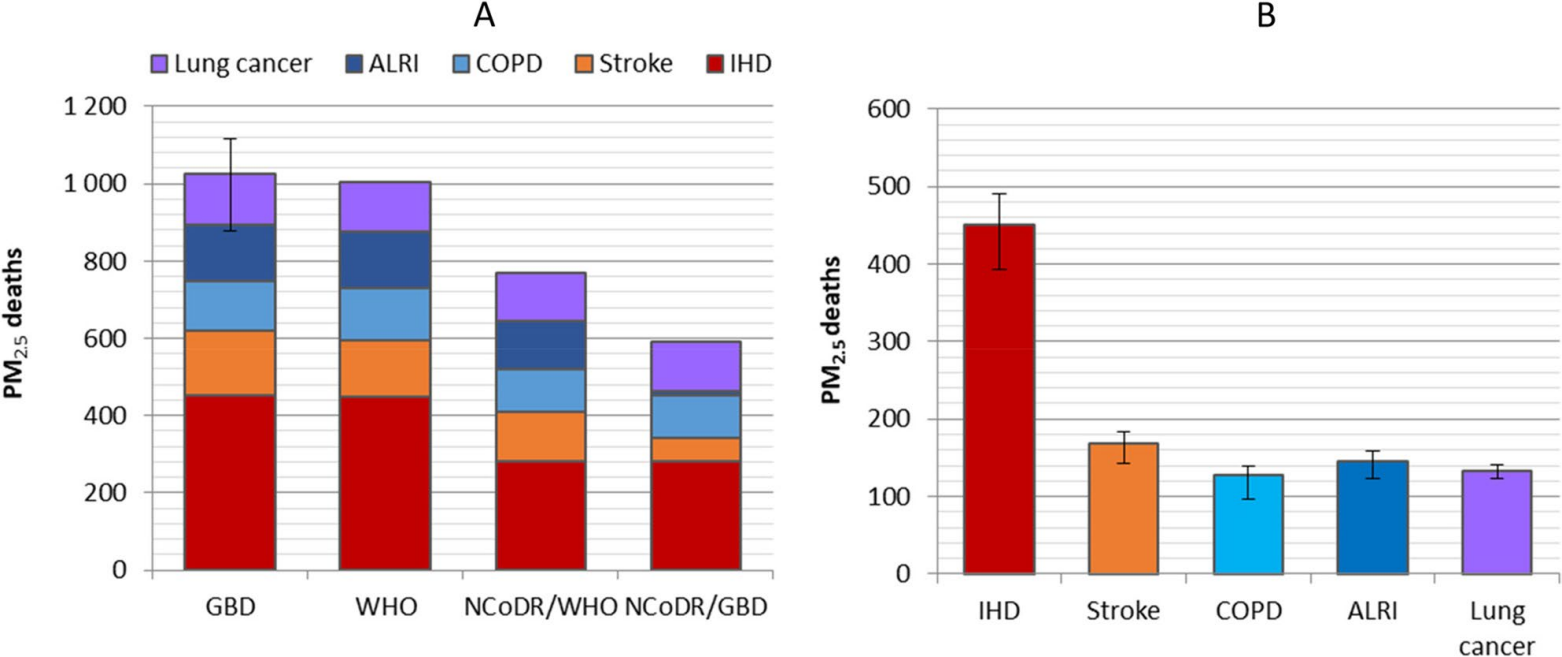
Influence of population health data sources on estimated deaths attributable to PM_2.5_ in Norway 2016. The figures show the influence of (**A**) different population health data sources from GBD, WHO and the Norwegian Cause of Death Registry (NCoDR) with different selections of ICD10 codes and (**B**) uncertainty intervals based on the 95% CI for the GBD disease-specific death estimates.

For all-cause mortality, the estimated number of deaths attributable to PM_2.5_ was very similar across the population health data sources and deviated less than 1% from the estimate of 1448 deaths based on the reference input data. In comparison, the estimated number of deaths based on disease-specific mortality varied more between the data sources, in line with the large differences observed in the input data for disease-specific deaths (Supplementary file 1,Table S2). The sum of five causes of death was 2% lower for WHO data, and 25% and 42% lower when Norwegian Cause of Death Registry data were used with WHO and GBD definitions, respectively, compared to the reference estimate of 1025 deaths. Notably, the estimates for stroke and ischemic heart disease were lower for Norwegian Cause of Death Registry data in comparison to the global GBD and WHO data sources (Fig. 8 A). The influence of uncertainties from the GBD population health data differed between the diseases, with the lowest uncertainties for lung cancer and ischemic heart disease (Fig. 8B, Supplementary file 1; Figure S3).

### Comparison across input data and methodological choices

At low exposure levels, the choice of relative risk had the largest impact, leading to variation between − 22% and 104% relative to the reference (Table 1). At a mean concentration of 5 µg/m^3^, applying a cut-off level between 2 and 6 µg/m^3^ reduced the estimated deaths by 39% to 91%, respectively, while using a log-log curve shape increased the estimates by about 40% compared to linear or log-linear shape. The influence of curve shape, cut-off and choice of exposure distribution was similar for all-cause and disease-specific mortality.

For exposure data, the influence of using GBD data led to a 39% increase compared to the national uEMEP model. For the population health data, the application of data from the Norwegian Cause of Death Registry resulted in a 42% reduction in attributable death estimates for the sum of five diseases compared to GBD data. However, for all-cause mortality, the differences between data sources were minor.

The largest uncertainty in attributable deaths was due to the 95% CI of relative risks, leading to combined differences of − 49% to 200% for all-cause mortality and − 61% to 69% for disease-specific mortality relative to the reference estimates. Combined uncertainty ranges from uEMEP and GBD exposure models were − 19% to 69% relative to the reference. For population health data, the uncertainty range for disease-specific mortality was − 42% to 9% across data sources (uncertainty estimates only for GBD). Uncertainties for all-cause mortality were negligible (−0.5% to 0.8%).

For the high exposure analysis (PWC 30 µg/m^3^) applying a cut-off between 2 and 6 µg/m^3^ reduced the estimated deaths by only − 5% to −17%, in contrast to low exposure level (PWC 5 µg/m^3^). Curve shape had the opposite influence at high exposures and log-log curve shape resulted in a −46% lower estimate compared to applying a linear curve. The choice of a relative risk at high exposure led to differences between − 19% and 53%.

### Influence on DALY estimates

The DALYs attributable to PM_2.5_ were estimated to be 11,730 (95% CI 5,980 − 16,790) using Chen & Hoek [2] cause-specific relative risks. The estimates were 32% lower (7,960 DALYs, 95% CI 5,310 − 10,540) with the MR-BRT curves and 31% higher (15,380 DALYs, 95% CI 13,120 − 17,630) with the GEMM curves. The uncertainty ranges reflect uncertainty from relative risks.

**Fig. 9:**
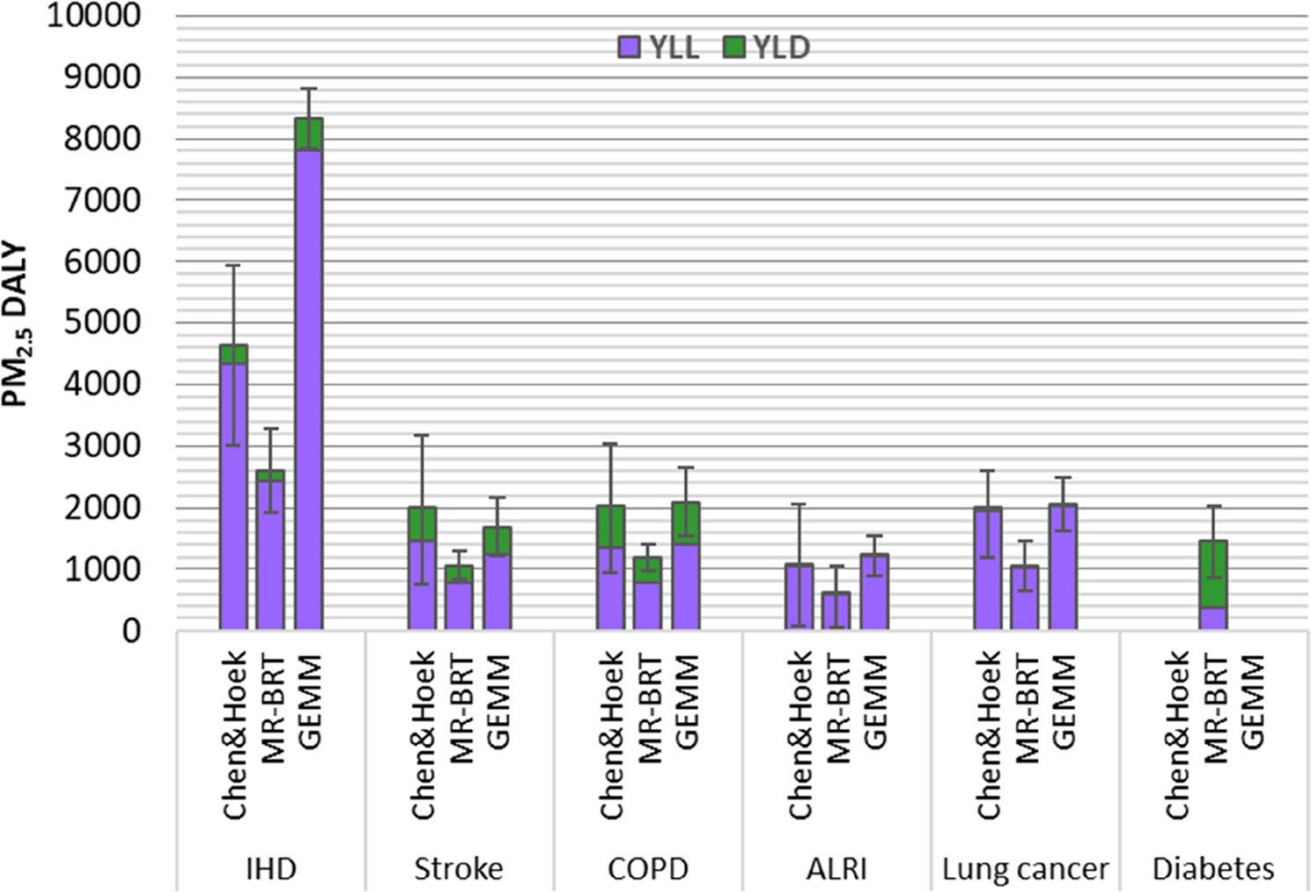
Burden of disease attributable to PM_2.5_ in disability-adjusted life years (DALY) for ischemic heart disease (IHD), stroke, chronic obstructive pulmonary disease (COPD), acute lower respiratory infections (ALRI), lung cancer and diabetes in Norway 2016. The columns show total DALYs divided in years lived with a disability (YLD) and years of life lost (YLL). DALYs are estimated using Chen & Hoek [2], MR-BRT, and GEMM concentration-response curves. Uncertainty bars reflect the 95% CI for relative risks for DALYs.

The estimated DALYs were predominantly driven by mortality (YLL), accounting for 67% to 94% of DALYs, except for diabetes, where morbidity (YLD) represented 74% of the total DALYs. Diabetes was only included in the MR-BRT curves (Fig. 9). Moreover, the choice of the risk estimate had a major influence on the disease-specific DALY estimates, a particularly large difference was found for IHD, where the MR-BRT and GEMM estimates were 44% lower and 79% higher than the reference, respectively. Note that the presented uncertainty intervals are based on the 95% confidence intervals of the concentration-response curves. Uncertainties in population health data were larger for YLD estimates than for YLL estimates [38].

## Discussion

This study quantified and compared the influence of methodological choices and data sources in health risk assessment of PM_2.5_ exposure, using Norway as a case study. Methodological choices related to the concentration-response curve had the greatest impact, with the choice of relative risk, cut-off, and curve shape resulting in changes of up to 104%, −91% and 40%, respectively, in attributable death estimates. In comparison, the choice of exposure data resulted in a 39% difference in attributable death estimates, while the source of population health data for disease-specific deaths contributed to up to a 42% variation. Uncertainties based on the 95% confidence intervals of the relative risks and population exposure distribution were wider than uncertainties for the population health data. Finally, DALY estimates for PM_2.5_ based on the GEMM curves were almost double the estimates based on the MR-BRT curves from the GBD, with the largest difference observed for ischemic heart disease.

The choice of relative risk estimate had the largest influence on the attributable death estimates. Using the relative risk from analyses restricted to exposures below 10 µg/m^3^ from Chen and Hoek [2] resulted in a doubling of death estimates. Several studies have reported higher risk estimates when analyses are restricted to exposures below 10–12 µg/m^3^ (e.g., [2, 6, 8]). However, effect estimates for restricted and full exposure ranges may not be directly comparable due to possible differences in population characteristics and particle composition at low and high PM_2.5_ levels [8].

Nevertheless, the large influence of choice of risk estimate on the attributable death estimates underlines the importance of choosing risk estimates based on high-quality studies, and with relevance for the geographical area, population and exposure levels under assessment. While the influence of relative risk at high exposure levels was smaller than at low exposures, it still significantly influenced the attributable burden estimates. More epidemiological studies are needed at low exposure levels to explore why the relative risks tend to be higher for low than for high PM_2.5_ exposures.

The attributable death estimates for PM_2.5_ based on all-cause mortality were generally higher than for disease-specific mortality, consistent with previous findings where non-accidental deaths were reported to be 30% higher than the sum of five disease-specific deaths [24]. Although this was partly due to the lower mortality rates for the five diseases combined, compared to all-cause mortality, the authors also suggested that PM_2.5_ exposure may be linked to mortality from causes beyond the five diseases considered [24]. In general, it is reasonable to assume that some causes of death classified as “all-cause” or “non-accidental” are not causally associated with exposure to PM_2.5_, and that those attributable deaths reflect some extent of overestimation. Moreover, both cause-specific morbidity and mortality estimates are necessary to estimate DALYs attributable to PM_2.5_. Thus, disease-specific risk estimates should be utilized when estimating DALYs. Currently, most epidemiological studies on PM_2.5_ focus on mortality, and more longitudinal studies on disease incidence are required to allow for a more accurate estimation of the morbidity burden.

While increasing the cut-off from 0 to 6 ug/m^3^ resulted in up to 91% reduction in attributable death estimates at low exposure levels (5 µg/m^3^), the corresponding reduction was only 17% for exposure distributions shifted to higher levels (30 µg/m^3^). Thus, the choice of cut-off is of particular importance for countries with low exposure levels. In line with our results, introducing a cut-off at 5 µg/m^3^ in the 2022 calculations of EEA for PM_2.5_ caused a higher reduction in estimates for exposure levels closer to the cut-off [15]. Globally, approximately 20% of the population are exposed to PM_2.5_ levels under 10 µg/m^3^, while 6% are exposed to levels below 5 µg/m^3^ [43].

Weichenthal et al. [5] estimated an additional 1.55 million deaths attributable to PM_2.5_ exposure globally after refining the shape of the concentration-response function at low exposures (2.5 to 5 µg/m^3^) for the Fusion model [44]. This corresponds to an approximately 17% increase in death estimates. The underestimation was larger in countries with low exposures. However, in absolute numbers, the underestimation in regions with annual exposure above 12 µg/m^3^ is larger since most of the people in the world are exposed to higher levels. Burnett et al. [45] compared global PM_2.5_ death estimates from four models and found that the GEMM and log-linear models produced the highest estimates, while IER yielded the lowest estimates, with the Fusion model falling in between.

Several arguments have been proposed for the choice of cut-off, including the lowest achievable concentration in the absence of anthropogenic sources (i.e., ‘natural’ background) or populated areas (i.e. ‘urban’ background), or the uncertainty in the risk estimates, i.e. the level above which health effects can be measured with confidence [24]. The reasoning underlying the choice of cut-off is important for interpreting the resulting estimates. In the comparative risk assessment framework of the GBD study, the theoretical minimum-risk exposure level (TMREL) reflects the lowest achievable exposure levels and is an even distribution between 2.4 and 5.9 µg/m^3^ [16]. In comparison, the EEA cut-off at 5 µg/m^3^ was chosen as it reflects the WHO Air Quality Guidelines, which recommend limit values for air quality at levels above which adverse effects are observed. In calculations for countries with low exposure levels, it may be feasible to consider lower cut-off levels, for instance, based on the national ‘urban background’ levels or the lowest levels of population exposure within the country. However, extrapolation of the concentration-response curve below the exposure levels reported in the underlying epidemiological studies introduces additional uncertainty.

The influence of the concentration-response curve shape was opposite for low and high-exposure distributions (mean 5 µg/m^3^ vs. mean 30 µg/m^3^), with a 41% increase and 50% decrease in attributable deaths, respectively, for the log-log curve compared to the linear curve. Most cohort studies report a linear or supra-linear association between annual PM_2.5_ exposure and the various mortality outcomes (e.g. [2, 8, 9, 46–48]). The log-log curve [29] applied in our study, has a supra-linear shape flattening out at higher concentrations. To our knowledge this is the only supra-linear single relative risk curve suggested or applied in health risk assessments of PM_2.5_. However, although it flattens out at higher concentrations, in line with the shape of the concentration-response curves fitted to cohort data, it appears to flatten too much at high concentrations compared to GEMM and MR-BRT curves. Thus, for certain purposes, a linear curve shape may be a feasible alternative when performing a health risk assessment. Alternatively, non-linear curves like GEMM and MR-BRT could be used. These are based on meta-regression of studies that are distributed across the entire exposure range. Their flexible functions allow for different curve shapes for each health outcome, based on the underlying data from the individual epidemiological studies.

The different sources of population health data, either the global data sources (GBD and WHO) or the national registry, resulted in very similar all-cause death estimates. However, for disease-specific deaths the differences were substantial, particularly for IHD, stroke and lower respiratory tract infections. The differences are most likely related to the fact that the GBD and WHO include a correction for ill-defined causes of death i.e. garbage codes, in their modeling of cause-specific deaths [39]. Mikkelsen et al. [49] found that in six high-income countries, the proportion of garbage codes in the death statistics varied from 22% to 36%. Garbage codes can distort the distribution of the true underlying causes of death, and addressing the garbage codes in data processing can reveal different mortality patterns [39]. In Norway, redistributing garbage codes led to significant changes in the ranking of causes of death, with the number of deaths due to IHD, COPD and stroke increasing by 35%, 23% and 316%, respectively, after correction (Supplementary file 2, Table S7). However, since the percent difference in number of deaths between Norwegian Cause of Death Registry and GBD does not mirror the percent change due to redistribution of garbage codes (Supplementary file 2, Table S8), the differences in the number of disease-specific deaths between the two data sources seem to be influenced by other aspects of the modelling as well. In countries with a high percentage of garbage codes in national registry data, future health risk assessments should consider using modelled data or implementing garbage code correction of registry data for disease-specific estimates. However, it should be noted that most epidemiological studies underlying the concentration-response curves are based on mortality data that have not been corrected for garbage codes, potentially creating a methodological inconsistency that warrants further research.

Regarding the exposure data, using the GBD model resulted in a 39% higher attributable death estimate compared to using data from the national uEMEP model. The GBD model is data-driven, with land-based measurements serving as a primary data source, and spatial distribution through a combination of satellite data and chemical transport model (CTM) calculations. This distribution is modelled at a resolution of 0.1⁰ x 0.1⁰ (equivalent to 11 km x 11 km at the equator), which is significantly lower than the 100 × 100 m^2^ resolution used by uEMEP. There are very few measurement sites in Norway, with most of them placed in the most polluted regions, i.e. within cities. The GBD model is therefore likely biased towards higher concentrations. In contrast, uEMEP is source-driven, using spatially distributed emissions at a 100 m resolution, allowing for more variability within a typical GBD-sized grid region. There can be up to a two-fold difference in concentrations within such a grid region as local sources often have a large influence, especially in populated areas [32]. Although uEMEP has a slight negative bias (−6%), this is minor compared to the overall differences between the two models. It is often observed, e.g. in Li et al. [17] and Korhonen et al. [20], that increasing the resolution of CTMs for exposure calculations leads to higher exposure estimates because emission sources for PM_2.5_ are often spatially correlated with population size. However, for data-driven models, the opposite can occur as shown by Bai et al. [50], where observed concentrations may represent much smaller regions than the grid sizes used in the model. It should also be noted that uncertainties in exposure data and exposure misclassification in epidemiological studies can impact the resulting risk estimates and may contribute to underestimation of the risk [51].

Uncertainties based on the 95% confidence intervals for relative risks and population exposure were wider than those for the population health data. Thus, when choosing one data source to cover overall uncertainty in reporting attributable deaths or disease burden, it is preferable to use the 95% confidence interval for the relative risks or population exposure. However, an integrated approach that combines the uncertainties from all input data is more advantageous. Approaches such as the Bayesian approach used by GBD or Monte Carlo simulation used in other studies [52, 53] offer this, although they are more computationally complex. A systematic review of the burden of disease studies in European countries found that, of 54 studies, only half reported uncertainty intervals [54]. The most reported uncertainties were related to morbidity or mortality outcomes (93%), and relative risks (82%), with the least reported being those related to exposure (62%).

The disease-specific MR-BRT curves used in the GBD study are primarily derived from cohort studies on mortality, although longitudinal studies on morbidity are included when available. The disease-specific GEMM curves and meta-analyses performed by Chen and Hoek for the revision of the WHO Air Quality Guidelines focus solely on mortality. GBD applies mortality-based curves to both morbidity and mortality calculations, reasoning that several longitudinal studies support an association between exposure and both disease incidence and mortality [55–57]. We rely on the same reasoning to support our use of mortality-based curves in the calculation of YLD. Although the DALY estimates were dominated by the YLL component, there is still need for more longitudinal studies assessing the association between PM_2.5_ exposure and morbidity. EEA took a different approach in their first assessment of morbidity attributable to PM_2.5_, using different risk estimates for mortality and morbidity [19].

Our results demonstrate that methodological choices and selection of data sources are of major importance for the resulting attributable death and DALY estimates for PM_2.5_. This is also reflected in the published estimates for deaths attributable to PM_2.5_ in Norway, which vary from 159 to 3230 deaths (Supplementary file 1, Table S4). The lowest estimate was based on the concentration-response curve reported by Chen & Hoek and a cut-off at 5 µg/m^3^ [28], and the highest estimate was based on the GEMM concentration-response curves [58]. In the methodological choices and selection of data sources, it is however important to consider the quality of the data source, including the accuracy, representativeness, and spatial resolution, not just the size of the resulting burden of disease estimate. Moreover, methodological development over time is likely to result in more accurate estimates. Thus, future health risk assessments should incorporate the latest developments in both data selection and methodology, as well as sensitivity analyses including a selection of input data to demonstrate the range of estimates resulting from methodological choices and selection of data sources.

The importance of input data selection in PM_2.5_ health risk assessments has gained increasing recognition in recent years (e.g.[3, 5, 45, 59]). However, to our knowledge, this study is the first to assess the influence of choices for all major input data, including exposure model, population health data, and several aspects related to the concentration-response function. This comprehensive approach allowed for a comparison of the magnitude of influence across the methodological choices and major input data sources. The results demonstrated that the choice of cut-off and relative risk had the greatest impact on the estimated deaths. Moreover, correcting the cause of death registry data for garbage codes, as done by GBD and WHO, contributes considerably to improved disease-specific attributable death estimates.

Although our results are specific to Norway, the methodological findings are broadly applicable to other countries with low PM_2.5_ exposure levels. Moreover, the analysis of high-exposure scenarios demonstrates that the importance of methodological choices extends beyond low-exposure settings. As the scientific basis for health risk assessment of PM_2.5_ continues to evolve, this presents challenges for stakeholders and researchers in formulating specific recommendations. Nevertheless, as pointed out by Castro et al. (2022), international collaboration is crucial for developing updated guidelines for health risk assessments, particularly in terms of consistency in reporting methodological choices and applied data. We recommend that the recently published Standardised Reporting of Burden of Disease Studies (STROBOD) guideline [60], is supplemented with a similar guideline for reporting of attributable burden of disease for environmental exposures.

## Conclusions

Our study demonstrates how methodological choices and different data sources influence the burden of disease estimates of PM_2.5_, especially for areas with low exposure levels (< 10 µg/m^3^). The results indicate that the burden estimates are highly sensitive to the selection of a cut-off, curve shape, and relative risk estimate. This sensitivity is reflected in the wide variability in the previously published estimates for deaths attributable to PM_2.5_. Our findings emphasize the importance of careful methodological considerations and transparent reporting of the methodological choices and data sources in PM_2.5_ health risk assessments to improve comparability and facilitate interpretations of the burden estimates.

## Supplementary Information

Supplementary File 1.

Supplementary File 2.

## Data Availability

The datasets used and/or analysed during the current study are available from the corresponding author on reasonable request.

## Abbreviations

AB: Attributable burden
ALRI: Acute Lower Respiratory Infections
AQG: Air Quality Guidelines
BoD: Burden of Disease
CI: Confidence Interval
COPD: Chronic Obstructive Pulmonary Disease
CTM: Chemical Transport Model
DALY: Disability-Adjusted Life Years
EEA: European Environmental Agency
EF: Exposed population Fraction
EPA: Environmental Protection Agency
GBD: Global Burden of Disease
GEMM: Global Exposure Mortality Model
IER: Integrated Exposure-Response
IHD: Ischemic Heart Disease
LRI: Lower acute Respiratory Infections
MR-BRT: Meta-Regression with Bayesian priors, Regularization and Trimming
NAAQS: National Ambient Air Quality Standards
NCD: Non-Communicable Diseases
NCoD: Norwegian Cause of Death Registry
PAF: Population Attributable Fraction
PM2.5: Fine particulate matter with aerodynamic diameter smaller than 2.5 µm
PWC: Population-Weighted Mean Concentration
RMSE: Root Mean Squared Error
RR: Relative Risk
STROBOD: Standardised Reporting of Burden of Disease Studies
TMREL: Theoretical Minimum-Risk Exposure Level
WHO: World Health Organization
YLD: Years Lived with Disability
YLL: Years of Life Lost
