# Supplementary material for: Burden of disease attributable to PM2.5 at low exposure levels: impact of methodological choices
Source: Environ Health. 2025 Dec 11;25:4. doi: 10.1186/s12940-025-01250-y (PMC12802007; doi:10.1186/s12940-025-01250-y)
Supplement: Supplementary file 1 [file 12940_2025_1250_MOESM1_ESM.docx]

# Supplementary file 1: Additional figures and tables

**Burden of Disease Attributable to PM_2.5_ at Low Exposure Levels: Impact of Methodological Choices**

Heli Lehtomäki^1,2*^, Gunn Marit Aasvang^3^, Gerhard Sulo^4^, Bruce Rolstad Denby^5^, Otto Hänninen^1^, Anette Kocbach Bølling^3^

**Affiliations**

^1^ Finnish Institute for Health and Welfare (THL), Health Security, Environmental Health, Helsinki, 00300, Finland 
^2^ University of Eastern Finland (UEF), Faculty of Health Sciences, School of Pharmacy, 70701 Kuopio, Finland

^3^ Department of Air Quality and Noise, Division of Climate and Environmental Health, Norwegian Institute of Public Health, Oslo, Norway

^4^ Department of Global Public Health and Primary Care, Section for Epidemiology and Statistics, University of Bergen, Norway

^5^ The Norwegian Meteorological Institute, Henrik Mohns Plass 1, 0313, Oslo, Norway

**Table of Contents**

Table S1. Relative risk (RR) estimates for PM_2.5_ exposure applied in this study.

**Table S2.** Population death data for Norway from different data sources.

Table S3. Population burden of disease estimates for Norway from GBD2019 for 2016.

Figure S1. Constructed exposure distributions.

**Figure S2.** Impact of relative risk (RR) estimates on deaths attributable to PM_2.5_ for high exposure levels.

**Figure S3.** Five causes of deaths attributable to PM_2.5_ in Norway in 2016.

**Table S4.** Comparison of estimated deaths attributable to PM_2.5_ estimated for Norway in different assessments.

**Table S5.** Impact of choices in concentration-response function to high and low exposure levels.

**References**

# Additional figures and tables

Table S1. Relative risk (RR) estimates for PM_2.5_ exposure applied in this study.

| **Cause** | **Relative risk (95% CI) per 10 µg/m^3^** | **Study type** | **Reference** |
| --- | --- | --- | --- |
| All-cause mortality^a^ | 1.14 (1.04-1.26)^b^ | Pooled analysis^e^ | Beelen et al. [1] |
| All-cause mortality^a^ | 1.06 (1.04-1.08) | Meta-analysis^e^ | Hoek et al. [2] |
| All-cause mortality^a^ | 1.08 (1.06-1.09) | Meta-analysis^e^ | Chen & Hoek [3] |
| All-cause mortality^a^ | 1.17 (1.12-1.23)^c^ | Meta-analysis^e^ | Chen & Hoek [3] |
| NCD +LRI | GEMM^d^ | Meta-analysis^f^ | Burnett et al., 2018 |
| IHD | 1.16 (1.10-1.21) | Meta-analysis^e^ | Chen & Hoek [3] |
| Stroke | 1.11 (1.04-1.18) | Meta-analysis^e^ | Chen & Hoek [3] |
| COPD | 1.11 (1.05-1.17) | Meta-analysis^e^ | Chen & Hoek [3] |
| ALRI | 1.16 (1.01-1.34) | Meta-analysis^e^ | Chen & Hoek [3] |
| Lung cancer | 1.12 (1.07-1.16) | Meta-analysis^e^ | Chen & Hoek [3] |
|  |  |  |  |
| IHD, stroke, COPD, ALRI, lung cancer | GEMM^d^ | Curve fitting^f^ | Burnett et al. [4] |
| IHD, stroke, COPD, ALRI, lung cancer, diabetes | MR-BRT^d^ | Curve fitting^f^ | GBD2019 Collaborative Network [5] |

^a^ excluding violent or accidental causes of deaths; ^b^ Hazard ratio (HR), transformed from the reported HR per 5 µg/m^3^; ^c^ combined estimate for five studies below 10 μg/m^3^; ^d^ relative risk (RR) depends on exposure level, ^e^ analysis assuming linear concentration-response curve, ^f^curve fitting of non-linear functions.

NCD + LRI: non-communicable diseases + lower respiratory infections; ALRI: Acute lower respiratory infection, COPD: Chronic obstructive pulmonary disease, IHD: Ischemic heart disease,

Table S2. Population death data for Norway from different data sources. Note that the population health data from WHO were only available for 2015 while the remaining data are from 2016. The difference between number of deaths between 2015 and 2016 was negligible within GBD2019. The numbers in brackets reflect percent difference from GBD 2019.

| Cause | GBD2019 | WHO 2015 | Norwegian registry GBD definitions | Norwegian registry WHO definitions |
| --- | --- | --- | --- | --- |
| All-cause mortality | 37,935 | 37,794 (0%) | 37,796 (0%) | 37,796 (0%) |
| NCD+LRI | 37,278 | 37,007 (-1%) | 27,232 (-27%) | 35,048 (-6%) |
| COPD | 2,468 | 2,605 (6%) | 2,159 (-13%) | 2,162 (-12%) |
| IHD | 6,130 | 6,103 (0%) | 3,850 (-37%) | 3,850 (-37%) |
| Stroke | 3,241 | 2,836 (-12%) | 1,132 (-65%) | 2,417 (-25%) |
| LRI | 1,983 | 1,972 (-1%) | 138 (-93%) | 1,676 (-15%) |
| Lung cancer | 2,376 | 2,318 (-2%) | 2,258 (-5%) | 2,240 (-6%) |
| Sum of 5 causes | 16,198 | 15,834 (-2%) | 9,537 (-41%) | 12,345 (-24%) |

NCD: non-communicable diseases, COPD: Chronic obstructive pulmonary disease, IHD: Ischemic heart disease, LRI: lower respiratory infections

Table S3. Population burden of disease estimates for Norway from GBD2019 for 2016.

| Cause | DALY | YLL | YLD | Deaths | YLD/DALY | YLL/death |
| --- | --- | --- | --- | --- | --- | --- |
| All-causemortality | 1,086,629 | 554,146 | 532,483 | 37,935 | 49 % | 14.6 |
| NCD+LRI | 1,062,139 | 546,327 | 515,811 | 37,278 | 49 % | 14.7 |
| COPD | 51,959 | 34,836 | 17,123 | 2,468 | 33 % | 14.1 |
| IHD | 84,313 | 78,982 | 5,331 | 6,130 | 6 % | 12.9 |
| Stroke | 51,629 | 37,860 | 13,768 | 3,241 | 27 % | 11.7 |
| LRI | 19,242 | 18,981 | 261 | 1,983 | 1 % | 9.6 |
| Lung cancer | 47,258 | 46,486 | 772 | 2,376 | 2 % | 19.6 |
| Diabetes | 31,823 | 8,275 | 23,548 | 603 | 74 % | 13.7 |

DALY: disability-adjusted life years, YLL: Years of life lost, YLD: Years lived with a disability, NCD: non-communicable diseases, COPD: Chronic obstructive pulmonary disease, IHD: Ischemic heart disease, LRI: lower respiratory infections


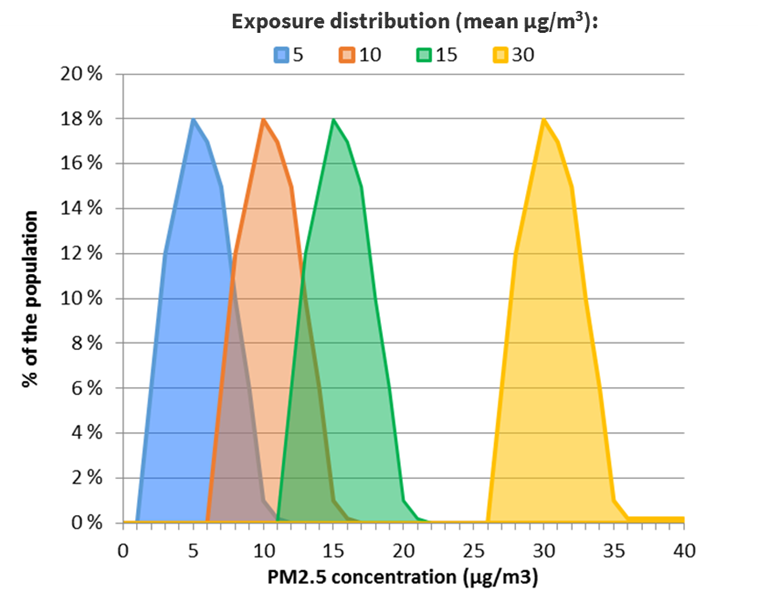


Figure S1. Constructed exposure distributions.


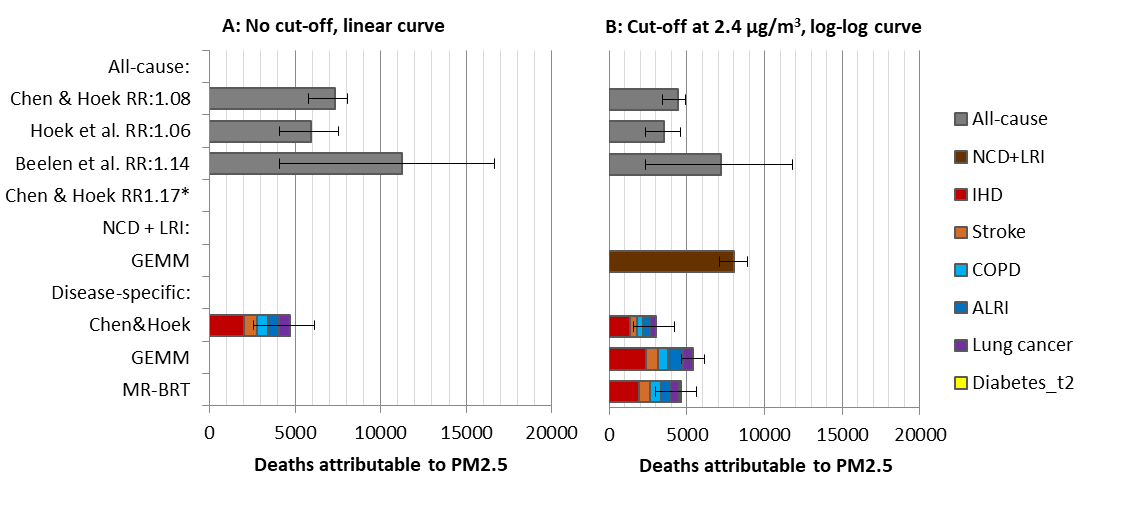


Figure S2 Impact of relative risk (RR) estimates on deaths attributable to PM_2.5_ for high exposure levels (mean PWC 30 µg/m^3^) with 95% CI from relative risks. For the disease specific estimates, uncertainty bars reflect the sum of uncertainties of the individual causes of death. In figure A linear concentration-response curve shape with no cut-off is assumed. In Figure B, the RRs for all-cause and disease-specific mortality were estimated using log-log curve with a cut-off at 2.4 µg/m^3^.NCD+LRI: Non-communicable diseases + lower respiratory.


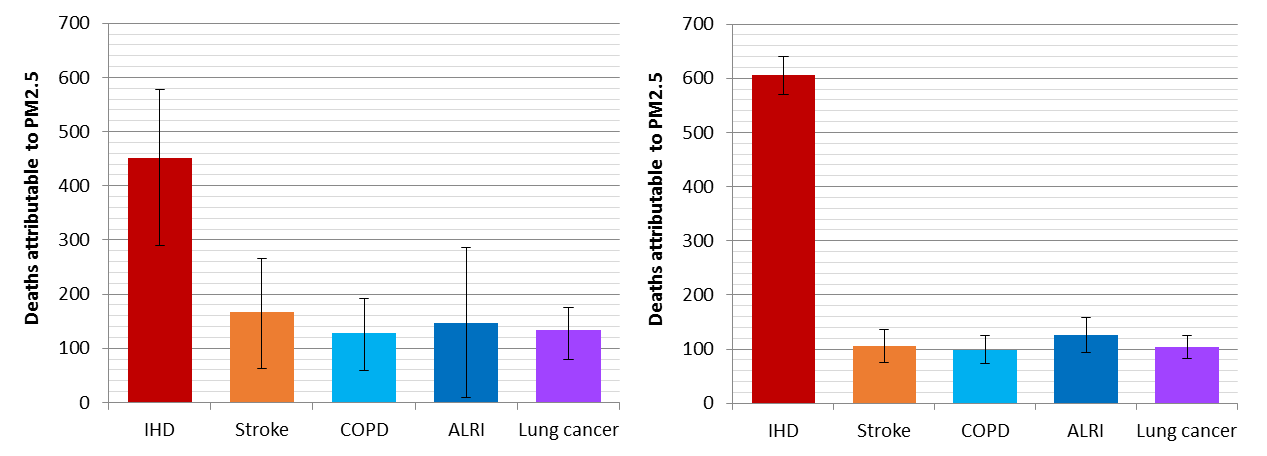


Figure S3. Five causes of deaths attributable to PM_2.5_ in Norway in 2016 estimated with A) Chen & Hoek [3] curves with 95%CI from relative risks and B) GEMM [4]. For GEMM curves a cut-off at 2.4 µg/m^3^ is applied but no cut-off is applied for Chen & Hoek [3] curves. Linear curve shape was assumed for Chen & Hoek [3] and non-linear for GEMM functions.

Table S4. Comparison of estimated deaths attributable to PM_2.5_ estimated for Norway in different assessments.

| Assessment | PM_2.5_ deaths | Baseline year | Mean exposure | Concentration-response curve | Cut-off  (µg/m3) | Health endpoints | Reference |
| --- | --- | --- | --- | --- | --- | --- | --- |
| GBD2017 | 967 (661-1265) | 2016 | 7 µg/m3 | IER | 2.4-5.9 | IHD, stroke, COPD, ALRI, lung cancer | GBD2017 [6] |
| GBD2019 | 408 (167, 690) | 2016 | 6.6 µg/m3 | MR-BRT | 2.4-5.9 | IHD, stroke, COPD, ALRI, lung cancer, diabetes, low birthweight, short gestation | GBD2019 [7] |
| WHO2016 | 636 (24, 1226) | 2012 | 9 µg/m3 | IER | 5.9-8.7 | IHD, stroke, COPD, ALRI, lung cancer | WHO [8] |
| EEA2019 | 1300 | 2016 | 5.9 µg/m3 | Hoek et al. [2] | 0 | All-cause mortality | EEA [9] |
| Lelieveld et al. | 3230 | 2015 | not available | GEMM | 2.4 | NCD+LRI? | Lelieveld et al. [10] |
| EEA2022 | 159 | 2020 | 4.6 (PWC), mean 2.3 | Chen & Hoek [3] | 5 | All-cause mortality | Soares et al. [11] |
| EEA2022 | 1031 | 2020 | 4.6 | Chen & Hoek [3] | 0 | All-cause mortality | Soares et al. [11] |

**Table S5.** **Comparison of impacts of relative risk (RR) estimates on deaths attributable to PM_2.5_ for low (PWC 5 µg/m^3^) and high exposure (PWC 30 µg/m^3^) levels.** Confidence intervals are presented for 95% CI from the relative risk.

|  |  | **Health endpoint** | | | | | | | |
| --- | --- | --- | --- | --- | --- | --- | --- | --- | --- |
| **PWC 5 µg/m^3^** | **C-R function characteristics** | **All-cause**  **(95% CI)** | **Disease-specific mortality (95%CI)** | | | | | |  |
|  |  |  | **IHD** | **Stroke** | **COPD** | **ALRI** | **Lung cancer** | **Diabetes** | **Sum** |
|  | **Linear, no cut-off** |  |  |  |  |  |  |  |  |
|  | Chen & Hoek [3]: total | 1448 (1097-1621) | 451 (290-578) | 168 (63-266) | 128 (60-192) | 146 (10-286) | 133 (80-175) |  | 1025 (502-1497) |
|  | Chen&Hoek[3]:<10 µg/m^3^ | 2950 (2131-3885) |  |  |  |  |  |  |  |
|  | Beelen et al.[1] | 2464 (738-4334) |  |  |  |  |  |  |  |
|  | Hoek et al. [2] | 1332 (738-1500) |  |  |  |  |  |  |  |
|  | **Log-log, 2.4 µg/m^3^ cut-off** |  |  |  |  |  |  |  |  |
|  | Chen & Hoek [3]: total | 1092 (829-1222) | 338 (218-432) | 126 (48-199) | 96 (45-144) | 109 (7-212) | 100 (60-131) |  | 769 (379-1117) |
|  | Chen&Hoek[3]:<10 µg/m^3^ | 2208 (1602-2895) |  |  |  |  |  |  |  |
|  | Beelen et al.[1] | 1848 (559-3223) |  |  |  |  |  |  |  |
|  | Hoek et al. [2] | 855 (559-1131) |  |  |  |  |  |  |  |
|  | GEMM | 1876 (1642-2109)^a^ | 605 (570-641) | 106 (76-136) | 99 (73-126) | 126 (93-158) | 103 (82-125) |  | 1040 (894-1185) |
|  | MR-BRT |  | 190 (140-238) | 66 (52-82) | 56 (46-67) | 61 (6-110) | 53 (33-73) | 28 (16-38) | 454 (293-608) |
| **PWC 30 µg/m^3^** | **Linear, no cut-off** |  |  |  |  |  |  |  |  |
|  | Chen & Hoek [3]: total | 7342 (5787-8065) | 1988 (1415-2369) | 804 (347-1136) | 612 (322-833) | 643 (58-1001) | 629 (412-771) |  | 4676 (2554-6111) |
|  | Beelen et al.[1] | 11220 (4065-16623) |  |  |  |  |  |  |  |
|  | Hoek et al. [2] | 5949 (4065-7563) |  |  |  |  |  |  |  |
|  | **Log-log, 2.4 µg/m^3^ cut-off** |  |  |  |  |  |  |  |  |
|  | Chen & Hoek [3]: total | 4421 (3502-7216) | 1303 (872-1620) | 501 (198-758) | 382 (186-551) | 422 (32-745) | 396 (245-505) |  | 3004 (1534-4180) |
|  | Beelen et al.[1] | 7216 (2322-11789) |  |  |  |  |  |  |  |
|  | Hoek et al. [2] | 3502 (2322-4571) |  |  |  |  |  |  |  |
|  | GEMM | 7997 (7090-8877)^a^ | 2356 (2244-2465) | 798 (595-986) | 613 (465-751) | 993 (794-1158) | 657 (537-770) |  | 5418 (4635-6129) |
|  | MR-BRT |  | 1886 (1454-2263) | 761 (609-907) | 683 (571-787) | 577 (64-901) | 551 (359-723) | 170 (107-222) | 4627 (3164-5803) |

^a^ GEMM all-cause deaths refer to deaths estimated for non-communicable diseases and lower respiratory infections (NCD+LRI)

**References**

1. Beelen, R., Raaschou-Nielsen, O., Stafoggia, M., Andersen, Z. J., Weinmayr, G., Hoffmann, B., ... & Vineis, P. (2014). Effects of long-term exposure to air pollution on natural-cause mortality: an analysis of 22 European cohorts within the multicentre ESCAPE project. The Lancet, 383(9919), 785-795.
2. Hoek G, Krishnan RM, Beelen R, Peters A, Ostro B, Brunekreef B, Kaufman JD (2013) Long-term air pollution exposure and cardio-respiratory mortality: a review. Environ Health 12, no. 1: 43.
3. Chen, J., & Hoek, G. (2020). Long-term exposure to PM and all-cause and cause-specific mortality: A systematic review and meta-analysis. Environment international, 105974.
4. Burnett, R., Chen, H., Szyszkowicz, M., Fann, N., Hubbel, B., Pope III, C.A., Apte, J.S., Brauer, M., Cohen, A., … & Spadaro, J.V. (2018). Global estimates of mortality associated with long-term exposure to outdoor fine particulate matter. PNAS, 115 (38) 9592-9597. <https://doi.org/10.1073/pnas.1803222115>.
5. GBD Collaborative Network. GBD 2019 Results. Seattle, United States: Institute for Health Metrics and Evaluation (IHME), 2020. Available from <https://vizhub.healthdata.org/gbd-results/>. Accessed 27 March 2023.
6. GBD 2017 Risk Factor Collaborators. Global, regional, and national comparative risk assessment of 84 behavioural, environmental and occupational, and metabolic risks or clusters of risks for 195 countries and territories, 1990–2017: a systematic analysis for the Global Burden of Disease Study 2017. Lancet 2018; 392: 1923–45.
7. GBD 2019 Risk Factors Collaborators. Global burden of 87 risk factors in 204 countries and territories, 1990–2019: a systematic analysis for the Global Burden of Disease Study 2019. The Lancet. 17 October 2020. doi:10.1016/S0140-6736(20)30752-2.
8. WHO. (2016). Ambient Air Pollution: A Global Assessment of Exposure and Burden of Disease;World Health Organization, 2016. Available online: <http://apps.who.int/iris/bitstream/10665/250141/1/9789241511353-eng.pdf?ua=1> (accessed 29 June 2023).
9. EEA (2019). Air quality in Europe—2019 report. European Environment Agency. <https://www.eea.europa.eu/publications/air-quality-in-europe-2019>. Accessed 30 June 2023.
10. Lelieveld J, Klingmüller K, Pozzer A, Burnett RT, Haines A, Ramanathan V. Effects of fossil fuel and total anthropogenic emission removal on public health and climate. Proc Natl Acad Sci U S A. 2019 Apr 9;116(15):7192-7197. doi: 10.1073/pnas.1819989116. Epub 2019 Mar 25. PMID: 30910976; PMCID: PMC6462052.
11. Soares, J., González Ortiz, A., Gsella, A., Horálek, J., Plass, D. & Kienzler, S. (2022). Health risk assessment of air pollution and the impact of the new WHO guidelines (Eionet Report – ETC HE 2022/10). European Topic Centre on Human Health and the Environment. <https://www.eionet.europa.eu/etcs/all-etc-reports>.
