# Supplementary material for: Burden of disease attributable to PM2.5 at low exposure levels: impact of methodological choices
Source: Environ Health. 2025 Dec 11;25:4. doi: 10.1186/s12940-025-01250-y (PMC12802007; doi:10.1186/s12940-025-01250-y)
Supplement: Supplementary file 2 [file 12940_2025_1250_MOESM2_ESM.docx]

# Supplementary file 2: Additional information regarding population health data

**Burden of Disease Attributable to PM_2.5_ at Low Exposure Levels: Impact of Methodological Choices**

Heli Lehtomäki^1,2*^, Gunn Marit Aasvang^3^, Gerhard Sulo^4^, Bruce Rolstad Denby^5^, Otto Hänninen^1^, Anette Kocbach Bølling^3^

**Affiliations**

^1^ Finnish Institute for Health and Welfare (THL), Health Security, Environmental Health, Helsinki, 00300, Finland 
^2^ University of Eastern Finland (UEF), Faculty of Health Sciences, School of Pharmacy, 70701 Kuopio, Finland

^3^ Department of Air Quality and Noise, Division of Climate and Environmental Health, Norwegian Institute of Public Health, Oslo, Norway

^4^ Department of Global Public Health and Primary Care, Section for Epidemiology and Statistics, University of Bergen, Norway

^5^ The Norwegian Meteorological Institute, Henrik Mohns Plass 1, 0313, Oslo, Norway

This additional file contains additional information regarding the ICD-10 codes used to extract numbers of disease specific death from NCoDR (table S5), the processing of mortality data under GBD and the comparison of the data for disease-specific deaths from GBD and NCoDR.

**Table of Contents**

**Section A1.** Comparison of health data from GBD, WHO and NCoDR.

**Table S6**. ICD-10 codes and intervals used by WHO GHE and GBD to define number of disease specific deaths.

**Table S7.** The impact of garbage code (GC) redistribution on the ranking of causes of death and the corresponding estimates.

**Table S8.** Comparison of number of disease specific deaths from NCoDR based on GBD definitions, and from GBD2019 data.

**References**

**Comparison of health data from GBD, WHO and NCoDR**

The ICD-10 codes used to extract data from Norwegian Cause of Death Register (NCoDR) [1] (table S5) were identical to the code list used by the respective organizations (GBD and WHO). Nevertheless, the number of disease specific deaths from NCoDR were considerably lower as compared to numbers from GBD and WHO. The difference between the GBD mortality and the corresponding NCoDR data are likely to be due to the processing of the mortality data under the GBD, and in particular the redistribution of garbage coded (GC) deaths.

The processing of mortality data in GBD is a five-step process [2]. First, all causes of death are mapped from their original coding onto the GBD cause list. Second, cause of death data inputs that are not available by detailed age and sex are split into the appropriate 105 categories. Third, deaths where the cause has been misclassified to other causes (e.g., Alzheimer’s disease and other dementias) are reassigned to the correct underlying cause. Fourth, deaths assigned to garbage code causes are redistributed (as illustrated for Norway in table S6). Fifth, misclassification of HIV-related deaths is corrected. Finally, before uploading of cause of death data to a central database for use in the GBD estimation process, noisy data due stochastic variation are smoothed.

For GBD 2019 study [3], the total percentage of GC deaths were around 17 %. The percent increase in the number of deaths after redistribution of GC deaths were 23% (COPD), 35% (IHD), 316% (stroke), 5682% (LRI) and 13% (lung cancer) (Supplementary table 6). The percent difference in number of deaths for Norway 2016 for NCoDR-GBD definitions vs. GBD 2019 is in the same order of magnitude as the percent change in death numbers due to redistribution of GC; 14 vs 23% (COPD), 59 vs. 35% (IHD), 186 vs. 316% (stroke), 1,337 vs 5682% (LRI) and 5 vs. 13% (lung cancer) (table S7). Thus, the difference between number of deaths reported by GBD 2019 and NCoDR based on GBD definitions is partly due to the redistribution of GC performed under the GBD processing of death data.

The difference in mortality data between GBD and WHO is likely to reflect the differences in ICD10 codes used by the two organizations (table S5). There may be differences in the specific steps of the processing of mortality data as well, but the similarity in data between the two organizations (table S2) points towards a similar processing.

**Table S6: ICD-10 codes and intervals used by WHO GHE and GBD to define number of disease specific deaths.** These disease definitions were used to request data from NCoDR for the aggregated number of deaths for ≥25y.

| **Health outcome** | **WHO GHE** [4] | **GBD**  **Causes of death [5]^[[1]](#footnote-1)^** |
| --- | --- | --- |
| **COPD** | J40-J44 | J41-J44.9 |
| **Stroke** | I60-I69 | G45-G46.8, I60-I63.9, I65-I66.9, I67.0-I67.3, I67.5-I67.6, I68.1-I68.2, I69.0-I69.3 |
| **IHD** | I20-I25 | I20-I25.9 |
| **Lung cancer** | C33-C34 | C33-C34.9, D02.1-D02.3, D14.2-D14.3, D38.1 |
| **LRI** | J09-J22, P23, U04 | A48.1, A70, B97.4-B97.6, J09-J15.8, J16-J16.9, J20-J21.9, P23.0-P23.4, U04-U04.9 |
| **NCD** | C00-C97, D00-D48, D55-D64 (minus D 64.9), D65-D89, E03-E07, E10-E34, E65-E88, F01-F99, G06-G98 (minus G14), H00-H61, H68-H93, I00-I99, J30-J98, K00-K92, L00-L98, M00-M99, N00-N64, N75-N98, Q00-Q99, X41-X42, X44, X45, R95 | A46-A46.0, A66-A67.9, B18-B18.9, B33.2, B86, C00-C13.9, C15-C25.9, C30-C34.9, C37-C38.8, C40-C41.9, C43-C45.9, C47-C54.9, C56-C57.8, C58-C58.0, C60-C63.8, C64-C67.9, C68.0-C68.8, C69-C75.8, C81-C86.6, C88-C96.9, D00.1-D00.2, D01.0-D01.3, D02.0-D02.3, D03-D06.9, D07.0-D07.2, D07.4-D07.5, D09.0, D09.2-D09.3, D09.8, D10.0-D10.7, D11-D12.9, D13.0-D13.7, D14.0-D14.3, D15-D16.9, D22-D27.9, D28.0-D28.7, D29.0-D29.8, D30.0-D30.8, D31-D36, D36.1-D36.7, D37.1-D37.5, D38.0-D38.5, D39.1-D39.2, D39.8, D40.0-D40.8, D41.0-D41.8, D42-D43.9, D44.0-D44.8, D45-D47.9, D48.0-D48.6, D49.2-D49.4, D49.6, D52.1, D55-D58.9, D59.0-D59.3, D59.5-D59.6, D60-D61.9, D63.1, D64.0, D66-D67, D68.0-D69.8, D70-D75.8, D76-D78.8, D86-D86.9, D89-D89.3, E03-E07.1, E09-E11.9, E15.0, E16.0-E16.9, E20-E34.8, E36-E36.8, E65-E68, E70-E85.2, E88-E89.9, F00-F03.9, F10-F16.9, F18-F19.9, F24, F50.0-F50.5, G10-G13.8, G20-G20.9, G23-G26.0, G30-G31.9, G35-G37.9, G40-G41.9, G45-G46.8, G47.3, G61-G61.9, G70-G73.7, G90-G90.9, G93.7, G95-G95.9, G97-G97.9, H05.0-H05.1, I01-I01.9, I02.0, I05-I09.9, I11-I13.9, I20-I25.9, I27.1, I28-I28.8, I30-I31.1, I31.8-I37.8, I38-I41.9, I42.1-I42.8, I43-I43.9, I47-I48.9, I51.0-I51.4, I60-I63.9, I65-I66.9, I67.0-I67.3, I67.5-I67.7, I68.0-I68.2, I69.0-I69.3, I70.2-I70.8, I71-I73.9, I77-I89.9, I95.2-I95.3, I97-I98, I98.2, I98.9, J30-J35.9, J37-J39.9, J41-J46.9, J60-J63.8, J65-J68.9, J70-J70.9, J82, J84-J84.9, J91-J92.9, J95-J95.9, K20-K29.9, K31-K31.8, K35-K38.9, K40-K46.9, K50-K52.9, K55-K62.9, K63.5, K64-K64.9, K66.8, K67, K68-K68.9, K70-K70.3, K71.7, K74-K74.9, K75.1-K75.2, K75.4-K76.2, K76.4-K77, K77.8, K80-K83.9, K85-K86.9, K90-K91.9, K92.8, K93.8-K95.8, L00-L05.9, L08-L08.9, L10-L14.0, L51-L51.9, L88-L89.9, L93-L93.2, L97-L98.4, M00-M03.0, M03.2-M03.6, M05-M09.8, M30-M36.8, M40-M43.1, M65-M65.0, M71.0-M71.1, M72.5-M72.6, M80-M82.8, M86.3-M86.4, M87-M87.1, M88-M89.0, M89.5, M89.7-M89.9, N00-N08.8, N10-N12.9, N14-N16.8, N18-N18.9, N20-N23.0, N25-N28.1, N29-N32.0, N32.3-N32.4, N34-N34.3, N36-N36.9, N39-N39.2, N41-N41.9, N44-N44.0, N45-N45.9, N49-N49.9, N60-N60.9, N65-N65.1, N72-N72.0, N75-N77.8, N80-N81.9, N83-N83.9, N84.0-N84.1, N87-N87.9, N99-N99.9, P04.3-P04.4, P70.2, P96.0-P96.2, P96.5, Q00-Q07.9, Q10.4-Q18.9, Q20-Q28.9, Q30-Q36, Q37-Q45.9, Q50-Q87.8, Q89-Q89.8, Q90-Q93.9, Q95-Q99.8, R50.2, R78.0-R78.5, R95-R95.9, X45-X45.9, X65-X65.9, Y15-Y15.9 |

Table S7. The impact of garbage code (GC) redistribution on the ranking of causes of death and the corresponding estimates. The table shows the ranked causes of death and the corresponding number of deaths for the top ten causes for Norway for 2015 for all ages before and after redistribution of garbage codes (GC). The table is based on Appendix figure 18 (page 176) in Johnson et al. [2]. Causes listed in bold reflect those included in the BoD estimates in the main paper.

| Before redistribution of GC | |  | After redistribution of GC | |
| --- | --- | --- | --- | --- |
| Cause of death (ranked) | Number of deaths |  | Cause of death (ranked) | Number of deaths  (% change) |
| **1. Ischemic heart disease** | **4213** |  | **1. Ischemic heart disease** | **5688 (35)** |
| 2. Alzheimers disease and other dementias | 3291 |  | 2. Alzheimers disease and other dementias | 3081 (-6) |
| **3. Tracheal, bronchus and lung cancer** | **2197** |  | **3. Chronic obstructive pulmonary disease** | **2587 (23)** |
| **4. Chronic obstructive pulmonary disease** | **2109** |  | **4. Tracheal, bronchus and lung cancer** | **2472 (13)** |
| 5. Colon and rectum cancer | 1606 |  | **5. Ischemic stroke** | **2093 (316)** |
| 6. Prostate cancer | 1046 |  | 6. Colon and rectum cancer | 1979 (23) |
| 7. Falls | 815 |  | 7. Falls | 1261 (55) |
| 8. Pancreatic cancer | 761 |  | 8. Prostate cancer | 1234 (18) |
| 9. Atrial fibrillation and flutter | 719 |  | **9. Other lower respiratory infections** | **983 (5682)** |
| 10. Breast cancer | 592 |  | 10. Pancreatic cancer | 893 (17) |
|  |  |  |  |  |
| **13. Ischemic stroke** | 503 |  | 12. Atrial fibrillation and flutter | 760 (6) |
| **101. Other lower respiratory infections** | 17 |  | 14. Breast cancer | 702 (19) |

Table S8. Comparison of number of disease-specific deaths from NCoDR based on GBD definitions, and from GBD 2019 data. The numbers of deaths were extracted from NCoDR based on the ICD-10 code definitions used by GBD, and from GBD 2019 study, respectively. The numbers correspond to those presented in table 2 in the main manuscript. The numbers in brackets reflect percent difference from NCoDR – GBD definitions, in contrast to table 2 that presents percent difference relative to GBD2019. The last column reflects the percent change in the GBD data for Norway after redistribution of garbage codes (these reflect the percentages presented in Supplementary table B), and are displayed for comparison between the difference between NCoDR-GBD definitions vs. GBD 2019, and percent change introduced during step 4 of the GBD processing of death data; the redistribution of garbage codes.

| Health endpoint | NCoDR -GBD definitions | GBD2019 |  | Percent change due to redistribution of GC in GBD 2019 |
| --- | --- | --- | --- | --- |
| COPD | 2,159 | 2,468 (14%) |  | 23% |
| IHD | 3,850 | 6,130 (59%) |  | 35% |
| Stroke | 1,132 | 3,241 (186%) |  | 316% |
| LRI | 138 | 1,983 (1,337%) |  | 5682% |
| Lung cancer | 2,258 | 2,376 (5%) |  | 13% |

COPD: Chronic obstructive pulmonary disease, IHD: Ischemic heart disease, LRI: lower respiratory infections

**References**

1. Norwegian Institute of Public Health. Dødsårsaksregisterets statistikkbank. Oslo; 2022. Norwegian. Available from: <http://statistikkbank.fhi.no/dar/>.
2. Johnson, S. C., Cunningham, M., Dippenaar, I. N., Sharara, F., Wool, E. E., Agesa, K. M., ... & Naghavi, M. (2021). Public health utility of cause of death data: applying empirical algorithms to improve data quality. BMC medical informatics and decision making, 21(1), 1-20.
3. GBD 2019 Risk Factors Collaborators. Global burden of 87 risk factors in 204 countries and territories, 1990–2019: a systematic analysis for the Global Burden of Disease Study 2019. The Lancet. 17 October 2020. doi:10.1016/S0140-6736(20)30752-2.
4. World Health Organization. WHO Methods and data sources for global burden of disease estimates 2000–2015. Geneva: World Health Organization; 2017. Available from: <https://www.who.int/healthinfo/global_burden_disease/GlobalDALYmethods_2000_2015.pdf?ua=1>
5. IHME. GBD 2017 Causes of Death and Nonfatal Causes Mapped to ICD Codes. Seattle, WA: IHME, University of Washington; 2019. Available from: <http://ghdx.healthdata.org/record/ihme-data/gbd-2017-cause-icd-code-mappings>

1. ICD-10 codes included in non-fatal burden of disease (YLD), differ from those included in number of deaths (YLL), but are not provided in this table. [↑](#footnote-ref-1)
